# Selective isolation and characterization of primary cells from normal breast and tumors reveal plasticity of adipose derived stem cells

**DOI:** 10.1186/s13058-016-0688-2

**Published:** 2016-03-12

**Authors:** Annika Weigand, Anja M. Boos, Kereshmeh Tasbihi, Justus P. Beier, Paul D. Dalton, Michael Schrauder, Raymund E. Horch, Matthias W. Beckmann, Pamela L. Strissel, Reiner Strick

**Affiliations:** Department of Plastic and Hand Surgery and Laboratory for Tissue Engineering and Regenerative Medicine, University Hospital of Erlangen, Friedrich-Alexander University of Erlangen-Nürnberg (FAU), Krankenhausstr. 12, Erlangen, D-91054 Germany; Department of Functional Materials in Medicine and Dentistry, University of Würzburg, Pleicherwall 2, Würzburg, Germany; Department of Obstetrics and Gynecology, University Hospital of Erlangen, Friedrich-Alexander University of Erlangen-Nürnberg (FAU), Erlangen, Germany

**Keywords:** Normal breast, Breast cancer, Stem cells plasticity, Primary cell lines, Tissue engineering

## Abstract

**Background:**

There is a need to establish more cell lines from breast tumors in contrast to immortalized cell lines from metastatic effusions in order to represent the primary tumor and not principally metastatic biology of breast cancer. This investigation describes the simultaneous isolation, characterization, growth and function of primary mammary epithelial cells (MEC), mesenchymal cells (MES) and adipose derived stem cells (ADSC) from four normal breasts, one inflammatory and one triple-negative ductal breast tumors.

**Methods:**

A total of 17 cell lines were established and gene expression was analyzed for MEC and MES (*n* = 42) and ADSC (*n* = 48) and MUC1, pan-KRT, CD90 and GATA-3 by immunofluorescence. DNA fingerprinting to track cell line identity was performed between original primary tissues and isolates. Functional studies included ADSC differentiation, tumor MES and MEC invasion co-cultured with ADSC-conditioned media (CM) and MES adhesion and growth on 3D-printed scaffolds.

**Results:**

Comparative analysis showed higher gene expression of *EPCAM, CD49f*, *CDH1 and KRTs* for normal MEC lines*;* MES lines e.g. *Vimentin, CD10, ACTA2* and *MMP9*; and ADSC lines e.g. *CD105*, *CD90*, *CDH2* and *CDH11*. Compared to the mean of all four normal breast cell lines, both breast tumor cell lines demonstrated significantly lower ADSC marker gene expression, but higher expression of mesenchymal and invasion gene markers like *SNAI1* and *MMP2*. When compared with four normal ADSC differentiated lineages, both tumor ADSC showed impaired osteogenic and chondrogenic but enhanced adipogenic differentiation and endothelial-like structures, possibly due to high *PDGFRB* and *CD34*. Addressing a functional role for overproduction of adipocytes, we initiated 3D-invasion studies including different cell types from the same patient. CM from ADSC differentiating into adipocytes induced tumor MEC 3D-invasion via EMT and amoeboid phenotypes. Normal MES breast cells adhered and proliferated on 3D-printed scaffolds containing 20 fibers, but not on 2.5D-printed scaffolds with single fiber layers, important for tissue engineering.

**Conclusion:**

Expression analyses confirmed successful simultaneous cell isolations of three different phenotypes from normal and tumor primary breast tissues. Our cell culture studies support that breast-tumor environment differentially regulates tumor ADSC plasticity as well as cell invasion and demonstrates applications for regenerative medicine.

**Electronic supplementary material:**

The online version of this article (doi:10.1186/s13058-016-0688-2) contains supplementary material, which is available to authorized users.

## Background

To this day, cell lines are pivotal for basic and clinical research and decisive for the analysis of gene/protein expression, signal transduction, pharmacological and toxicological experiments. Cell lines are the gold standard for laboratory analyses due to their ease of handling, self-replication, availability and homogenous nature [1]. This is true for breast cancer research, where a wealth of published data is based upon cell lines, however, most were derived from pleural effusion fluids of patients with advanced-stage cancer [2]. The heterogeneity of breast cancer, and the large variety of available breast cancer cell lines require a careful choice of optimal cell lines for specific experimentation [3]. In an analysis of 51 human breast cancer cell lines, recurrent genome aberrations and the resulting transcriptional changes mirrored those of breast tumors, although other substantial differences were detected [4]. Genotypical and phenotypical drift after a range of subcultures of cell lines is also quite frequent, thus contributing to discrepancies when comparing different studies [5]. Moreover, there are no cell lines for male breast cancer, several rare histopathological subtypes or uncommon chromosomal aberrations/mutations of breast cancer [3, 4].

Normal human breast tissue is composed of glands with a network of luminal epithelial, myoepithelial and progenitor cells embedded in a stroma, consisting of cells of mesenchymal lineage, adipocytes, fibroblasts, immune, blood and endothelial cells, including the extracellular matrix (ECM) essential for three-dimensional microstructure [6]. Breast myoepithelial and luminal epithelial cells stem from bipotent or other progenitor cells where myoepithelial cells could also form directly from the luminal lineage [7]. Interestingly, human breast cancer cells are mainly of luminal epithelial lineage, expressing mostly epithelial markers and the myoepithelial cell is thought to even have tumor suppressing properties [8]. According to newer cell classifications, human breast tissue contains two diverse luminal and two basal epithelial differentiation states that vary on the basis of CD24, epithelial cell adhesion molecule (EPCAM) and CD49f (Integrin alpha 6 or ITGA6) expression. It is postulated that human breast cancer tissues also contain these four cellular states, but in altered ratios compared to normal tissues [9].

In addition to established cell lines the use of primary cells isolated and grown in culture from human tissue also provides a powerful and representative tool for examining biological processes in vitro. In addition to the frequently poor availability of primary human tissue, the main barriers are the isolation procedure, cultivation and characterization of primary cells. If all these properties are in place, then cell functional studies can be performed to address specific questions. For example, studies have been performed to address the question of whether primary tumor stromal cells significantly influence the development of breast cancer [10]. Additionally, as the complexity of breast tumor development is based upon the fact that tumor and non-tumorigenic cells co-exist, primary cultures could address why tumor cells have different clonal evolution and plasticity, and the contribution of cancer stem and progenitor cells to the observed heterogeneity [11]. Interestingly, primary adipose-derived stem cells (ADSC) influence MCF-7 cells by increasing their proliferation when co-cultured [12, 13].

Primary cells are also currently implemented for tissue engineering and regenerative studies [14]. For example, to date, several types of breast augmentation for plastic surgery and reconstruction are available: on the one hand implants such as saline and silicone, on the other, autologous fat transfer (AFT). Saline and silicone implants do not support growth and vascularization and have a possible risk of leakage, capsule fibrosis and infection, and need multiple changes. In AFT the patient’s own fat tissue from other parts of the body is injected into the breast, however, the procedure often needs several rounds of grafting, and presently it is unknown how non-breast-fat stem cells differentiate in the breast [15]. Although there are recent reports of increasing numbers of ADSC injections into human breasts, there are no adequate data on the potential carcinogenic effects to the mammary epithelium. Taken together, to gain new insights into normal and tumor cell development, studying primary cell types in culture, or studying the interaction between different cell types derived from normal and tumor breast tissue, is currently possible [11].

Different isolation methods have been used to fractionate breast epithelial cells from normal tissue [16, 17] and tumor tissue for cultivation [18, 19]. These methods often involve time-consuming and expensive isolation steps, such as immunomagnetic or fluorescence-activated flow sorting, facilitating the selection of one distinct cell type from normal breast or tumor tissue or even from epithelial cell cultures [17, 20, 21]. However, in most studies isolation and characterization of only one specific cell type is performed. Therefore, in this present study we simultaneously isolated three different cell types including mammary epithelial (MEC), mesenchymal (MES) and ADSC from normal or tumor breast tissues from the same patients. Cells were further characterized and compared for the expression of specific epithelial, mesenchymal, endothelial and stem cell markers. Studies were also performed comparing ADSC differentiation into osteogenic, chrondrogenic, adipogenic, and endothelial-like cells from normal and tumor breast tissues. Importantly, we addressed the influence of conditioned media (CM) isolated from normal and breast cancer ADSC during adipocyte differentiation, on invasion of breast cancer MEC and MES cells in vitro. Last, we demonstrated an application involving primary breast cells for regenerative medicine.

## Methods

### Patients

Tissue samples were obtained from healthy women: normal mammary cells (NORMA1-4) (from women with mean age 37 years) in medically indicated cases or surgery to the breast. Samples were also obtained from two women with breast cancer (mean age 59.5 years), one with invasive inflammatory ductal carcinoma (IFDUC1) and one with triple-negative ductal carcinoma, respectively (TRIDUC1) (Table 1). Human tissue collection was approved by the Ethics Committee of the University of Erlangen-Nürnberg (Germany) (Ethics number 264_13B) in accordance with the World Medical Association Declaration of Helsinki. Informed consent was obtained from all patients.

**Table 1:** Tissues used for cell isolations from patients

	Origin	Side	Tumor	Age, years	ER	PR	her2	Ki67	Type
TRIDUC1	breast	left	yes	73	neg	neg	neg	70 %	basal cell
IFDUC1	breast	right	yes	46	pos	pos	1+	90 %	ductal
NORMA1	breast	left + right	no	32					
NORMA2	breast	left + right	no	23					
NORMA3	breast	right	no	43					
NORMA4	breast	left	no	50					

*ER* estrogen receptor, *PR* progesterone receptor, *her2* human epidermal growth factor receptor-2, *TRIDUC*1 triple-negative ductal carcinoma 1, *IFDUC1* invasive inflammatory ductal carcinoma 1, *NORMA1-4* normal mammary cells 1-4

### Isolation of the epithelial (MEC) and mesenchymal (MES) cell fractions

**Fig. 1:**
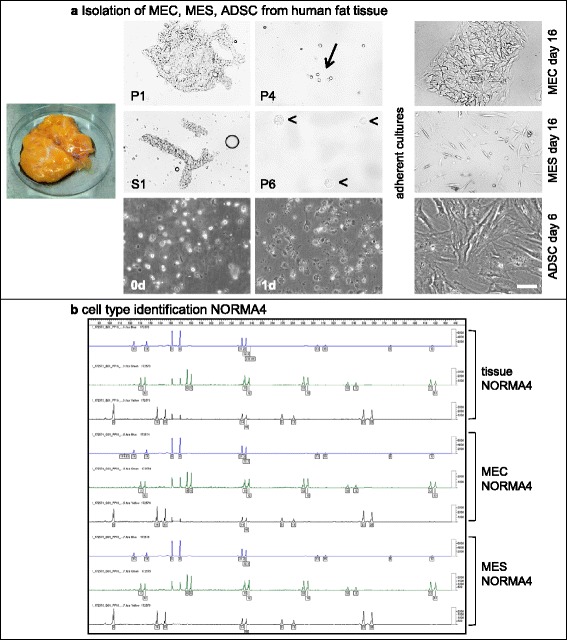
Isolation of mammary epithelial cells (*MEC*), mesenchymal cells (*MES*) and adipose-derived stem cells (*ADSC*) from human fat tissue and cell type identification of normal mammary cells (*NORMA*)4. **a** Breast epithelial and mesenchymal cells were isolated from normal and tumor tissue and ADSC were isolated from adipose tissue. Isolation of MEC and MES resulted in two critical fractions, a pellet (*P1*) and supernatant (*S1*), which were the basis for the fractionation of epithelial (*first row*) and mesenchymal cells (*second row*), respectively. The P1 fraction was enriched with large epithelial extralobular or intralobular duct-like glands and with further fractionation steps (P4) we observed enrichment of single epithelial cells, whereas the P6 was enriched with mesenchymal cells. The pre-coating of the cell culture dishes with collagen enhanced the attachment and proliferation of the epithelial cells, where cells grew exponentially up to 30 days as epithelial cell clusters. Immediately after isolation ADSC were seeded in cell culture flasks (*third row*, *first left*). At 24 h after isolation (*third row*, *middle*) over 95 % of rounded cells became adherent. Cellular morphology changed rapidly within the next few days to an elongated shape (*third row, right*). *Scale bar* 100 μm. **b** DNA fingerprinting used for cell line identification. NORMA4 primary breast tissue and the isolated MEC and MES primary cell lines had 100 % congruence for all 21 short tandem repeats (STRs). *Lines* on each graph represent STRs: *blue*, *green* and *black* indicate identities between the different samples

Up to 50 ml of fresh control or tumor breast tissue was isolated and processed in a sterile manner. However, different amounts of tissues can be processed accordingly. Tissues were washed extensively with 1 × PBS without centrifugation, incubated in 1 × PBS with 1 × penicillin/streptomycin (Life Technologies, Carlsbad, CA, USA) (1:1 tissue (ml) to 1 × PBS) for 1 h at room temperature (RT), cut into small pieces with removal of vascular material and then digested with 0.5 × collagenase/hyaluronidase (StemCell Technologies, Vancouver, BC, Canada) in DMEM plus 1 × penicillin/streptomycin enzymes according to Smith et al. and Emerman et al. [22, 23] but with some modifications. For 50 ml of tissue the digestion period was 16 h at 37 °C with orbital shaking at 75 rpm. Following digestion, cells were diluted 1:1 with 1 × PBS and then centrifuged using 88 g for 30 s at RT. The top fat layer (yellow) was discarded and the supernatant containing single mesenchymal cells (S1) was transferred into a 50-ml polypropylene tube and further purified later as described below. The remaining pellet (P1) contained large epithelial duct-like clusters (sometimes called organoids) derived from columnar epithelium (extralobular) or a double layer of cuboidal epithelium (intralobular) (Fig. 1a). P1 was further digested with 0.01 % Trypsin (Gibco/Life Technologies) in 1 × PBS for 30 minutes at 37 °C and 5 % CO_2_ on a rotator (Miltenyi Biotec GmbH, Bergisch-Gladbach, Germany) to further dissociate epithelial cells and any glandular-attached mesenchymal cells. For trypsin inactivation and further fractionation, 5 ml of Epicult media + 5 % FCS (EMF + 5 %) (StemCell Technologies) was added, the cells resuspended and then centrifuged at 88 g for 30 s at RT. The pellet fraction (P2), enriched of epithelial cells, was then washed with EMF + 5 % and centrifuged 217 g for 3 minutes at RT. In case of remaining residual undigested collagen fibers, 10 ml EMF + 5 % was added to the pellet (P3) and then passed through a 100-μm filter (BD, Franklin Lakes, NJ, USA). The flow-through, mainly enriched with single epithelial cells was then washed three times with EMF + 5 % and centrifuged 217 g for 3 minutes at RT. The pellet (P4) was resuspended in EMF + 5 % supplement C (StemCell Technologies) and then seeded on previously coated collagen (collagen R from Serva Electrophoresis GmbH, Heidelberg, Germany) tissue culture flasks (approximately 200,000 cells per 75 cm^2^ culture dish). After 24 h the medium was changed to serum-free EMF + 1 × supplement C and 0.5 μg/ml hydrocortisone (StemCell Technologies). Primary normal and tumor mammary epithelial cells (MEC) were grown short-term for up to 30 days without senescence (maximum eight cell passages). Note that primary MEC from TRIDUC1 were not isolated.

To obtain a purer mesenchymal cell fraction the supernatant (S1) was passed through a 40-μm filter (BD) to remove any small epithelial duct-like glands and centrifuged 217 g for 3 minutes at RT. The remaining pellet (P5) consisted of enriched mesenchymal cells and contaminating red blood cells (RBCs). For RBC lysis, the pellet P5 was resuspended in 1 ml of 1 × PBS and 3 ml RBC-lysis solution (0.8 % NH_4_Cl, 0.1 mM ethylenediaminetetraacetic acid (EDTA), pH: 8.0) on a rotator (Miltenyi) for 12 minutes at RT. The cells were then washed twice with 5 ml of EMF + 5 % at 217 g for 3 minutes at RT and the pellet (P6) was resuspended in EMF + 5 % plus 1 × supplement C and 0.5 μg/ml hydrocortisone. Approximately 200,000 cells were seeded per 75-cm^2^ culture dish previously coated with collagen R and incubated at 37 °C and 5 % CO_2_. Primary normal and tumor mammary mesenchymal cells (MES) were grown up to 30 days without senescence (maximum eight cell passages) (Fig. 1a).

### Isolation of adipose-derived stem cells (ADSC)

ADSC isolation was performed according to an adapted protocol of Bunnell et al. [24]. Approximately 30 ml of breast tissue was minced and digested with 0.1 % collagenase type I (Biochrom GmbH, Berlin, Germany) at 37 °C for 60–120 minutes. The enzyme digestion was stopped with minimum essential medium (MEM) alpha (Life Technologies) supplemented with 10 % FCS (FCS superior, Biochrom) and centrifuged at 400 g for 10 minutes. The cell pellet was incubated in RBC lysis buffer (17 mM tris-hydroxymethyl-aminomethane, 16 mM NH_4_Cl) for 10 minutes at RT. After centrifuging at 300 g for 10 minutes, the cell pellet was resuspended in MEM alpha with 10 % FCS and 1 × penicillin/streptomycin (Biochrom GmbH), filtered through a 100-μm and 70-μm mesh cell strainer (BD Biosciences, Franklin Lakes, NJ, USA) and seeded in cell culture flasks. The medium was changed two to three times a week. When reaching 80–90 % confluency, ADSC were split in a ratio 1:3 using accutase (400–600 units/ml, Sigma-Aldrich Corporation, St. Louis, MO, USA). Experiments were performed at passage 4–7. For characterization, ADSC were seeded on cell culture plates and differentiated into the osteogenic, chondrogenic, adipogenic, and endothelial-like lineages.

### DNA fingerprinting of original breast tissue and derived primary cell lines

For cell line identification we employed the analysis of short tandem repeats (STRs), normally used for forensic samples and paternity tests using the Powerplex®21 System (Promega, Corporation, Fitchburg, WI, USA). The analysis included the amplification and detection of all 13 combined DNA index system (CODIS) loci (D3S1358, D5S818, D7S820, D8S1179, D13S317, D16S539, D18S51, D21S11, CSF1PO, FGA, TH01, TPOX, vWA) and six loci of Amelogenin, Penta D, Penta E, D1S1656, D2S1338, D6S1043, D12S391 and D19S433. Genomic DNA (500 pg) of the original tissue and different established primary cell lines was isolated and purified. The PCR was performed with the Powerplex®21 System (Promega), the STR amplified, sequenced and visualized.

### Immunocytochemistry

For characterization of isolated cells, immunocytochemical staining was performed. Cells in passage 4–5 were seeded in 8-chamber culture slides (BD). GATA binding protein 3 (GATA-3) was visualized after fixation with 4 % formaldehyde and permeabilization with 0.25 % Triton-X-100. For reduction of background staining blocking with 10 % goat serum (Life technologies) was performed for 30 minutes. Cells were incubated with a mouse monoclonal GATA-3 antibody (1:100; IgG1, Zytomed Systems, Berlin, Germany) for 1 h at RT and Alexa Fluor® 488 goat anti-mouse IgG1 (γ1) antibody (1:200; Life Technologies) for 1 h at RT. For CD90 staining, cells were fixed with 4 % paraformaldehyde. After blocking with 5 % goat serum for 30 minutes, cells were incubated with anti-CD90 (1:200, mouse monoclonal (F15-42-1) IgG1, Acris antibodies, San Diego, CA, USA) for 1 h at RT. For visualization Alexa Fluor® 594 goat anti-mouse IgG1 (γ1) Antibody (1:200; Life Technologies) was used for 1 h at RT. After fixation of cells with 10 % formaldehyde and blocking with 5 % goat serum for 30 minutes, cells were stained with a monoclonal mouse anti-human cytokeratin (KRT) antibody (type 5, 6, 8, 17 and 19) (1:100; clone MNF116, Dako, Glostrup, Denmark) for 1 h at RT. For visualization the secondary antibody Alexa Fluor® 488 goat anti-mouse IgG1 (γ1) Antibody (1:500; Life Technologies) was used for 30 minutes at RT.

Cells were fixed with 4 % paraformaldehyde for detection of the epithelial membrane antigen (EMA)/MUC1. Background staining was reduced using 3 % H_2_O_2_. Staining was performed with a mouse monoclonal anti-EMA IgG1 (1:50; clone GP1.4, Abcam PLC, Cambridge, UK) for 1 h at 37 °C using the LSAB System HRP (Dako). Counterstaining was performed with hemalaun staining (Merck Millipore, Billerica, MA, USA) and 4',6-diamidino-2-phenylindole (DAPI) (1:1 000; Life Technologies). Evaluation of staining was performed with a microscope and a digital camera using computer-assisted image capture software (Olympus IX83, cellSens Software, Olympus Corporation, Tokio, Japan).

### Quantification of cytokeratin-positive cell fraction

Cell fractions of NORMA1-3 in passage 4–5 were seeded in 12-well chamber plates to calculate the percentage of KRT (type 5, 6, 8, 17 and 19)-positive cells within the different cell isolates. After fixation and KRT staining, counting of positive cells was performed automatically using the Olympus cellSens Count & Measure Software. The percentage of KRT-positive cells versus total cell amount was calculated.

### Real-time PCR

For RNA extraction cells in passage 4–5 were harvested with accutase (400–600 units/ml, Sigma-Aldrich). RNA of all probes was extracted using the RNeasy Mini Kit with corresponding QIAshredder Homogenizer (Qiagen, Hilden, Germany). RNA was reverse-transcribed into cDNA with QuantiTect Reverse Transcription Kit with a DNase I incubation (Qiagen). SsoAdvanced Universal SYBR Green Supermix (Bio-Rad Laboratories, Hercules, CA, USA) with a Light Cycler (Bio-Rad iCycler iQ5) was used for quantitative real-time PCR. All kits were used according to the manufacturers’ protocols. Samples were tested as triplicates. Tyrosine 3-monooxygenase/tryptophan 5-monooxygenase activation protein, zeta (*YWHAZ*) was used as a housekeeping gene. Data analysis was performed using the 2^-ΔΔCT^ method.

For *CD34,* control RNA human blood endothelial cells (BEC) (PromoCell GmbH, Heidelberg, Germany) was used. Control RNA from human peripheral blood cells (PBC) was used for *CD14, CD19, CD45* and *HLA-DR*. Lysis of erythrocytes was performed by incubation 1:10 in lysis buffer for 5 minutes at RT. After washing and centrifuging steps (300 g, 5 minutes) RNA was isolated as described for cell isolates. Additional file 1 specifies all primers used for real-time PCR.

### Differentiation of adipose-derived stem cells (ADSC) into four different cell lineages

The differentiation potential of ADSC derived from normal breast tissue (NORMA1-4) and tumor tissue (IFDUC1, TRIDUC1) into osteogenic, chondrogenic, adipogenic, and endothelial-like lineages was investigated. ADSC were seeded in 24-well plates and cultivated in MEM alpha supplemented with 10 % FCS for 3 days, and afterwards were incubated using differentiation conditions.

For osteogenic differentiation ADSC were cultivated in osteogenic induction medium consisting of MEM alpha (Life Technologies) supplemented with 1 μM dexamethasone, 10 mM β-glycerophosphate and 50 μM ascorbic acid (all from Sigma-Aldrich). After 28 days, cells were analyzed with Alizarin red (AppliChem GmbH, Darmstadt, Germany) indicating osteogenic differentiation.

Chondrogenic differentiation of ADSC was studied using cell pellets derived from a series of steps. First, a cell suspension of 1.5 × 10^6^ ADSC was diluted in 100 μl 2 % sodium alginate (Sigma-Aldrich) then the cell suspension was pipetted and added as drops into a 0.1 M CaCl_2_ solution and allowed to gel for 10 minutes at RT. After washing with 0.15 M NaCl and PBS the cell pellets were cultivated in hMSC chondrogenesis induction medium (Provitro GmbH, Berlin, Germany) for 28 days. Supplements essential for the chondrogenic induction media included recombinant human insulin, human transferrin, sodium selenite (ITS), dexamethasone, ascorbic-acid-2-phosphate, proline and the human growth factor TGF-β3 (all from Provitro GmbH) and were added to the basal medium. For detection of chondrogenic differentiation Alcian blue staining (Alcian blue 8 GX, Merck Millipore) was performed according to the manufacturer’s protocol.

For differentiation into adipocytes, ADSC were cultured in adipogenic induction medium (Provitro GmbH) for 21 days. The medium was supplemented with FCS, L-glutamine and HEPES, insulin, dexamethasone, indomethacine and 3-isobutyl-1-methyl-xanthine (all from Provitro GmbH). Lipid vacuoles were detected by the Oil Red O staining (Sigma-Aldrich) and counterstaining was performed with hemalaun (Mayer's hemalaun solution, Merck KGaA, Darmstadt, Germany).

The endothelial differentiation potential of ADSC was examined by performing a capillary-like tube formation assay. Each well of a μ-slide (ibidi GmbH, Planegg, Germany) was filled with 10 μl of Matrigel® (Growth Factor Reduced Basement Membrane Matrix, Corning Inc., Corning, New York, NY, USA), which was allowed to polymerize for 30 minutes at 37 °C. Matrigel® contains several growth factors like vascular endothelial growth factor (VEGF) (1.0–1.5 ng/ml) and basic fibroblast growth factor (bFGF) (0.0–0.1 pg/ml) (Corning Inc.: www.corning.com/media/worldwide/cls/documents/ CLS-DL-CC-026 DL.pdf) known to induce differentiation if the tested cell type has endothelial potential [25]. ADSC and human mammary epithelial cells (HMEC) were seeded onto the wells in triplicates at a density of 1 × 10^4^ cells/well and cultured in 50 μl MEM alpha containing 10 % FCS or mammary epithelial cell culture medium (Provitro GmbH), respectively, for 5 h.

Evaluation of cells using the different staining procedures was performed with a microscope and a digital camera using computer-assisted image capture software (Olympus IX83, cellSens Software, Olympus Corporation, Tokio, Japan). ImageJ (http://imagej.nih.gov) computer software was used to measure colony area sizes from differentiated cultures (osteogenic and chondrogenic), or the total number of single cells containing lipid droplets (adipogenic). The following parameters were quantified for endothelial-like capillary structures: 1) total length of each capillary side structure; 2) the area of all closed and non-closed capillary structures; and 3) the total number of closed capillary structures as loops using the Leica application suite V3 (Leica Microsystems, Wetzlar, Germany).

### Invasion analysis of breast cancer MEC and MES cells under the influence of conditioned media from different stages of ADSC adipocyte differentiation

Cell invasion assays were performed according to previous reports [26, 27]. Calf skin type I collagen G (Serva Electrophoresis GmbH, Heidelberg, Germany) and rat tail type I collagen R (Biochrom AG, Berlin, Germany) were mixed at a ratio of 1:1 plus 0.1 volume of sodium bicarbonate (23 mg/ml), 0.1 volume of 10 × DMEM, and then the solution was neutralized with sodium hydroxide. An aliquot of 0.6 ml was added to each 12-well plate and then polymerized to obtain a 0.6-cm collagen bed.

For cell culture 30,000 cells of IFDUC1 MEC (cultivated in mammary epithelial cell growth medium; Provitro GmbH containing 10 ng/ml epidermal growth factor (EGF), 0.5 μg/ml hydrocortisone, 5 μg/ml insulin and 4 % bovine pituitary extract (BPE)) or IFDUC1 MES (cultivated in Epicult media plus 5 % charcoal-treated serum (CTS) but with no supplement) were added to the collagen beds and incubated for 1 h at 37 °C to allow the cells to adhere. Conditioned media (CM) from IFDUC1 or NORMA4 ADSC cells grown at different stages of adipocyte differentiation (7, 14 and 21 days) were then added in a series of dilutions (1:10, 1:20 and 1:30) to IFDUC1 MEC and MES cells on collagen. After 72 h all cells were fixed in 4 % paraformaldehyde. All invaded cells were counted in >20 optical fields per well and represented as number of cells per cm^2^ according to previous reports [26, 27]. Criteria for mesenchymal and amoeboid phenotypes were as previously reported [28].

For the production of CM, ADSC-NORMA4 and ADSC-IFDUC1 were seeded in 6-well plates and cultivated in 3 ml MEM alpha + 10 % FCS until 80–90 % confluency. Cells were then cultivated in adipogenic differentiation medium supplemented with FCS, L-glutamine and HEPES, insulin, dexamethasone, indomethacine and 3-isobutyl-1-methyl-xanthine (all from Provitro GmbH). Two thirds of the medium were changed every 2 days and the same amount of CM was collected at time points 7, 14 and 21 days. To concentrate, CM was centrifuged in filter tubes (Amicon® Ultra-15 Centrifugal Filter Devices, 3 K; Merck KGaA, Darmstadt, Germany) at 4000 g for 30 minutes and stored at −80 °C until further use.

### Normal breast MES cell growth on a 3D printed scaffold substrate and microscopy

Poly(ε-caprolactone) (PCL) scaffolds (20 μm × 20 μm squares) were fabricated using melt electrospinning writing (MEW) as reported elsewhere [29]. The fibers were spaced 150 μm apart and deposited at 90° to each other, either as a single strand or layered 20 times. Prior to cell seeding, the PCL scaffolds were pre-treated with ethanol to remove air bubbles, hydrated with sterile deionized water, incubated with 1 M NaOH for 30 minutes at RT and washed several times with 1 × PBS. Each scaffold was then incubated with 10 μg/ml fibronectin isolated from human plasma (Roche Diagnostics, Rotkreuz, Risch, Switzerland) in 1 × PBS for 1 h at RT in a 35-mm tissue culture dish. A polydimethylsiloxane (PDMS) cast ring was placed on top of the scaffold to secure it in place: 250,000 primary fractioned MES cells (NORMA2) were added to the scaffolds in EMF + 5 % plus 1 × supplement C and 0.5 μg/ml hydrocortisone. Cells were monitored every 3 days using phase contrast microscopy (Carl Zeiss AG, Oberkochen, Germany) to document growth. After 18 days of growth when the cells reached confluence the cells were fixed with 4 % paraformaldehyde for 15 minutes at RT then washed once with 1 × PBS. Cells were stained with the wheat germ agglutinin Alexa Fluor 488 conjugate (Invitrogen/Life technologies) specific for membranes and DRAQ5 (Biostatus Ltd., Shepshed, UK) for cell nuclei according to manufactures’ instructions and then imaged using a confocal laser scanning microscopy (Leica, SP5X). Laser power was set to approximately 1.2 mW to avoid extensive photobleaching. Alexa was detected with an Argon laser at 488 nm and DRAQ5 with a He-Ne laser at 633 nm.

### Statistics

Data are expressed as the mean ± standard error of the mean. Statistical analysis was performed using SPSS 20.0 for Windows (SPSS Inc., Chicago, IL, USA). Results were interpreted statistically using one-way analysis of variance (ANOVA) and the Tukey honest significant difference (HSD) post-hoc test. The normal distribution was confirmed using the Shapiro–Wilk test. For non-normal data distributions, the non-parametric Kruskal–Wallis test and the Mann–Whitney *U* test were used. Results of gene expression in the individual patients were interpreted statistically using the Mann–Whitney *U* test. The level of statistical significance was set at *p* ≤0.05. A *p* value ≤0.01 was considered highly significant. All graphics were created using Microsoft Excel for Windows 2010.

## Results

### Isolation of mammary epithelial (MEC) and mesenchymal cells (MES) resulted in highly purified and stable primary cell lines

Following the initial overnight digestion of control breast or tumor tissue, cells were diluted and then centrifuged at a low speed and time (88 g for 30 s). This critical low speed and time resulted in two fractions, a pellet (P1) and supernatant (S1), which were the basis for the fractionation of epithelial and mesenchymal cells, respectively. The P1 fraction was enriched with large epithelial extralobular or intralobular duct-like clusters and with further fractionation steps (P2 and especially P3 and P4) we observed enrichment of single epithelial cells when cultured on collagen-coated dishes (Fig. 1a). The pre-coating of the cell culture dishes with collagen enhanced the attachment and proliferation of the epithelial cells, where cells grew exponentially up to and beyond 30 days as MEC clusters (Fig. 1a). Additionally, the epithelial phenotype remained stable in EMF medium, which is a serum-free, hormone enriched medium containing hydrocortisone.

The fractionation of mesenchymal cells began with the first supernatant (S1). Following several fractionation steps the remaining cell pellet (P6) yielded single cells with a mesenchymal phenotype when cultured on collagen-coated culture dishes. The mesenchymal cells grew exponentially up to and even beyond 30 days in the same EMF medium as above, but containing 5 % FCS (Fig. 1a). Therefore, from four different normal breast tissues and two breast tumors we successfully isolated and established MEC and MES primary cell lines.

Additionally, for future cell line identification, we implemented a DNA fingerprinting technique amplifying and sequencing 21 STR markers (Powerplex® 21 System) (Fig. 1b). Following the analyses of the original normal breast tissue (NORMA4) and the derived MEC and MES primary cell lines a 100 % identical match was found for all 21 STR markers. This result demonstrates that DNA fingerprinting is a powerful tool for verifying congruence with the original tissue, cell line identification and proof of cell line purity.

### Immunocytochemical analysis demonstrated a variety of different cell markers specific for cell types in normal and tumor cells

**Fig. 2:**
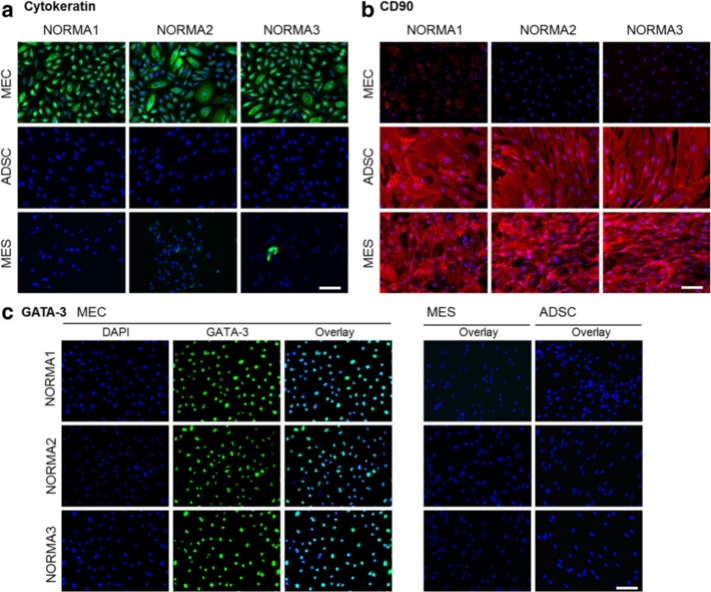
Immunofluorescence staining of cell isolates. **a** Immunofluorescence hybridization demonstrated the epithelial origin of cell isolates incubated with a pan-cytokeratin (KRT) type 5, 6, 8, 17 and 19 antibody. All epithelial cell fractions had positive localization of the cytoplasm (*green*). In contrast, in the mesenchymal cell fraction very few positive cells were identified (e.g., normal mammary cells (*NORMA*)3). Adipose-derived stem cells (*ADSC*) were KRT-negative. Nuclei were identified using 4',6-diamidino-2-phenylindole (DAPI) (*blue*). **b** CD90 immunocytochemical analysis was performed for mesenchymal cell characterization. Positive localization was visible in ADSC and mammary mesenchymal cell (*MES*) fractions (*red*). In contrast, epithelial cells were largely CD90-negative. Only in NORMA1 were a few rounded-shape cell colonies sparsely positive. Counterstaining was performed using DAPI (*blue*). **c** GATA-3 was visualized in epithelial cell isolates using immunocytochemical analysis. Almost all cells in the epithelial cell fraction expressed this transcription factor but it was not detected either in the MES or ADSC fraction (*green*). Counterstaining was performed using DAPI (*blue*). *Overlay* demonstrates co-localized cells with *turquoise* nuclei. *Bar* 100 μm

**Fig. 3:**
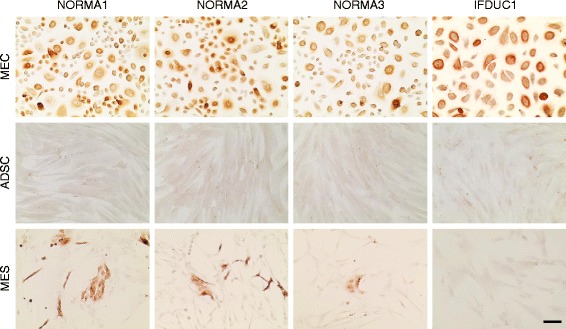
Epithelial membrane antigen (EMA)/MUC1 immunocytochemical staining of cell isolates. Mammary epithelial cells (*MEC*), adipose derived stem cells (*ADSC*) and mesenchymal cells (*MES*) primary cell lines all from the same patients (three normal and one breast-tumor primary cell lines) are indicated *above*. MEC had positive protein expression for EMA/MUC1 (*brown*). ADSC were negative for MUC1 protein expression. In the MES very few small colonies were identified as MUC1-positive. *Bar* 100 μm. *IFDUC* invasive inflammatory ductal carcinoma

To determine the purity of different cell types following isolation and cell culturing we implemented a variety of antibodies specific for different cell markers, which could distinguish epithelial and mesenchymal origins, including ADSC. For determination of epithelial cell origin, we used a cytokeratin (KRT) antibody, which could detect a variety of KRT types (5, 6, 8, 17 and 19). All MEC fractions had almost 100 % positive KRT staining of the cytoplasm (Fig. 2a). In contrast, in the ADSC fractions virtually no KRT-positive cells were found but on the other hand some MES cell fractions demonstrated very low percentages of KRT-positive cells (e.g., NORMA2 3.1 % and NORMA3 2.11 %). CD90 immunocytochemical analysis was performed for mesenchymal cell identification. Positive staining indicated high expression of the cell-surface glycoprotein CD90 in the spindle-shaped ADSC and MES fractions (Fig. 2b). In contrast, epithelial cells from all fractions were >95 % CD90-negative. Only NORMA1, had a few round-shaped cell colonies (about 10 %), which were sparsely positive. GATA-3, a member of the zinc finger transcription factor family, which plays an important role in cell proliferation and differentiation of luminal glandular epithelial cells in mammary glands, was visualized in epithelial cell isolates using immunocytochemical analysis. Over 95 % of epithelial cells from all fractions expressed this transcription factor in the nucleus; however, GATA-3 was not detected in MES or ADSC cultures (Fig. 2c). Additionally, we found that over 90 % of epithelial cells representing all fractions stained positively for the epithelial membrane antigen (EMA)/MUC1 (Fig. 3). In the MES fractions only a few colonies (<5 %) were MUC1-positive and in the ADSC fraction MUC1 was not detected (Fig. 3). Our findings support that each isolated and established cell line represents >95 % similarity with cell-type-specific markers.

### Characterization of ADSC, MEC and MES primary cell lines using real-time PCR

**Fig. 4:**
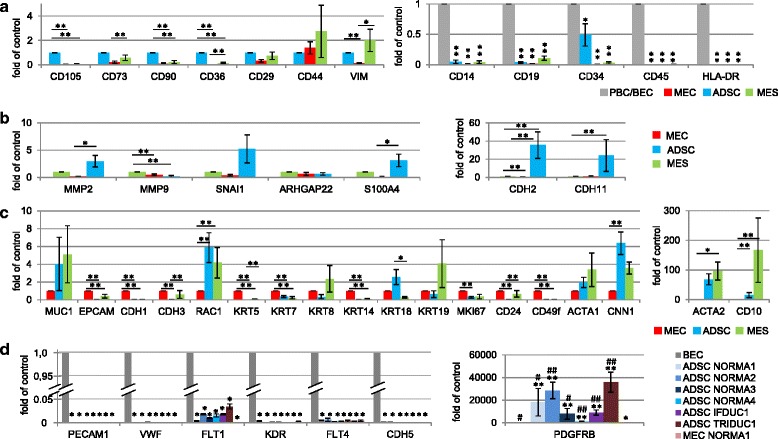
Gene expression of cell isolates. Real time PCR results show gene expression (*n* = 48) genes, including a house keeping gene after comparison to the mean expression of all normal (*NORMA*1-4) respective *MEC*, MES or ADSC primary cell lines. **a** Expression of positive stem cell markers *CD105*, *CD73*, *CD90*, *CD36*, *CD29*, *CD44* and of vimentin (*VIM*) (*ADSC*=1) and hematopoietic markers *CD14, CD19, CD34, CD45, HLA-DR* are shown (PBC/BEC=1). **b** Panel shows invasion and EMT marker genes, *MMP2, MMP9 and SNAI1,* the fibroblast mesenchymal marker S100A4 and N-cadherin (*CDH2*) and *CDH11*, markers of the mesenchymal phenotype (*MES*=1). **c** Epithelial cell markers (MEC=1) compared to ADSC and MES. Specific markers include epithelial stem cell markers *CD24* and *CD49f* and markers of basal myoepithelial mammary cells (*ACTA1, CNN1, ACTA2, CD10*). **d** Six endothelial marker genes analyzed were *PECAM1, VWF, FLT1, KDR, FLT4, CDH5* and *PDGFRB* (BEC=1). Significant values compared with BEC (^*^, ^**^) or MEC NORMA1 (^#^, ^##^). (^*^,^#^ p ≤ 0.05, ^**^,^##^ p ≤ 0.01) (ADSC = adipose derived stem cells, BEC = blood endothelial cells MEC = mammary epithelial cells, MES = mesenchymal cells, PBC = peripheral blood cells)

Following our cell-type protein expression analyses described above, we then profiled all primary cell lines (NORMA1-4) using real-time PCR for 48 genes to help further classify specific cell phenotypes. Compared to all MEC and MES fractions, ADSC fractions had increased expression of the cell surface markers *CD105, CD73, CD90, CD36* and *CD29,* where *CD105, CD90* and *CD36* expression were significantly higher (Fig. 4a, Additional file 2a-d). The intermediate filament protein vimentin (VIM), a well-known marker for mesenchymal cells, clearly demonstrated significantly higher levels of gene expression in MES when compared to MEC, but was not significantly different to ADSC (Fig. 4a, Additional file 2a-d). Furthermore, *N-cadherin (CDH2)* and *CDH11*, additional markers of the mesenchymal phenotype, were strongly expressed in ADSC compared to both MEC and MES primary cell lines (Fig. 4b, Additional file 3a-d). Finally, there was greater expression of the fibroblast mesenchymal marker *S100A4*, which is associated with a more differentiated state, in both MES and ADSC compared to MEC (Fig. 4b, Additional file 3a-d). Expression of a range of typical epithelial cell markers was investigated in all primary cell lines using real-time PCR (Fig. 4c, Additional file 4a-d). As expected, MEC expressed epithelial cell markers such as *MUC1*, *EPCAM*, *E-cadherin* (*CDH1*), *P-cadherin* (*CDH3*) and six cytokeratin genes (*KRT5/7/8/14/18/19*), supporting a general epithelial phenotype. However, expression of some markers, such as *MUC1* and the cytokeratin *KRT8* and *KRT19* genes expressed on luminal cells, varied between MES primary cell lines (Fig. 4c, Additional file 4a-d). Significantly greater expression of *RAC1*, known as a regulator for various cellular functions including differentiation of mammary epithelia, was observed in ADSC and MES compared to MEC, supporting different functions for this gene among primary breast cells (Fig. 4c).

We also examined other genes in all primary cell lines (NORMA1-4) derived from patients, which correlated with proliferation, stem cells and muscle-specific genes (Fig. 4c, Additional file 4a-d). For example, although all primary cell lines expressed the proliferation marker *MKI67*, there was significantly greater expression in MEC compared to the other primary cell lines. Additionally, there was significantly greater expression of the epithelial stem cell markers *CD24* and *CD49f* in MEC compared to MES and ADSC. Finally, there was significantly lower expression of markers of basal myoepithelial mammary cells, including *calponin (CNN1), alpha smooth muscle actin (ACTA2)* and *CD10*, in all MEC fractions, supporting there being no significant myoepithelial component when compared to ADSC and/or MES cell fractions. In contrast, our results point to a positive association between muscle characteristics (*CNN1* and *ACTA2*) and ADSC or MES.

Exploring the expression of key genes involved in invasion and signaling pathways, the matrix metalloprotease (*MMP)* 2 gene*,* specific for digestion of the ECM, was significantly greater in ADSC, when compared to MEC, however, there was no difference to MES (Fig. 4b, Additional file 3a-d). In contrast, expression of *MMP9* was significantly greater in MES when compared to ADSC and MEC (Fig. 4b, Additional file 3a-d). Interestingly, very low expression levels of *SNAI1*, a transcriptional repressor of *CDH1* expression and a marker of epithelial to mesenchymal transition (EMT), were associated with each MEC primary cell line when compared to the MES and ADSC. For signaling pathways, although the amoeboid cell shape indicator Rho GTPase activating protein 22 (*ARHGAP22*) gene was differentially expressed, no significant differences were observed between primary cell lines.

Last, we analyzed the presence of hematopoietic cell markers in all primary cell lines, and endothelial cell marker genes specifically in ADSC lines where, for example, *CD34* is associated with stem cells. We observed that there was significantly lower expression of *CD14, CD19, CD45* and *HLA-DR*, with the exception of *CD34,* on all representative cell types when compared to a positive control consisting of PBC or BEC (Fig. 4a, Additional file 2g-j). These results demonstrate no contamination of peripheral blood cells in the primary cell lines. While there was significantly lower expression of *CD34* in MEC and MES normal and tumor primary cell lines compared to blood cells, interestingly, ADSC *CD34* expression was in general significantly greater among primary cell lines (Fig 4a). Although the expression of six endothelial differentiation marker genes (*PECAM1*, *vWF*, *FLT1*, *FLT4*, *KDR*, *CDH5*) was significantly lower in normal and tumor ADSC, there was significantly greater expression of *PDGFRB* in ADSC when compared to BEC and NORMA1 MEC cells (Fig. 4d). These results support the sharing of the progenitor *CD34* and *PDGFRB* expression found in endothelial cells and pericytes, which is necessary for angiogenesis [30, 31], in both normal and tumor ADSC.

### Characterization and comparison of breast tumor primary cell lines (IFDUC1, TRIDUC1) with NORMA1-4 breast primary cell lines

**Fig. 5:**
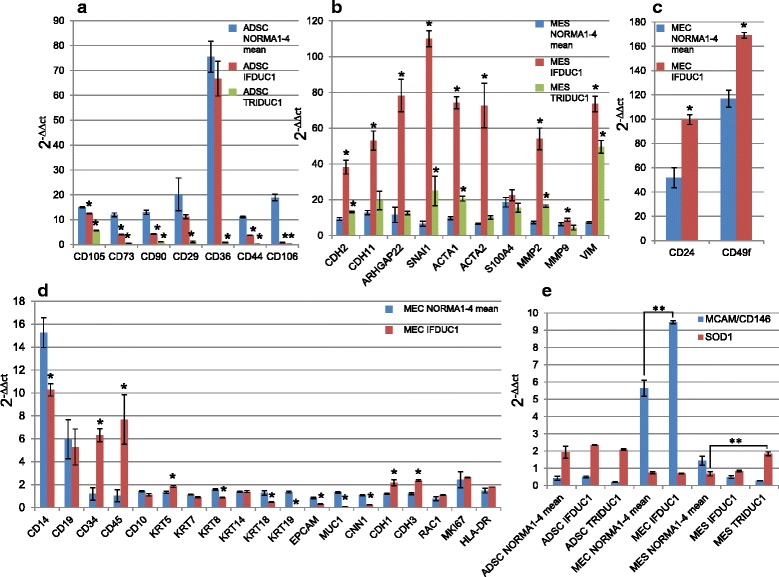
Gene expression results of cells derived from cancer tissue compared to normal breast tissue. Gene expression comparison of adipose-derived stem cell (*ADSC*), mesenchymal cell (*MES*) and mammary epithelial cell (*MEC*) primary cell lines of invasive inflammatory ductal carcinoma (*IFDUC1*) and triple-negative ductal carcinoma (*TRIDUC1*) with the representative primary cell lines of normal mammary cells (*NORMA*)1-4. Real-time PCR results were calculated for 2^-∆∆ct^ (*Y-axes*) for the marker genes of ADSC (**a**), MES (**b**) and MEC (**c**, **d**). For MEC primary cell lines only IFDUC1 was compared with NORMA1-4 (MEC mean). **e** Gene expression levels of two novel breast cancer markers, *CD146/MCAM* and *SOD1* 2^-∆∆ct^ (*Y-axes*) comparing both breast cancer cell lines with NORMA1-4. For each cell line the NORMA1-4 values were averaged

We found significant differences when comparing the gene expression of cell fractions isolated from the tumors TRIDUC1 or IFDUC1 with the mean of each gene from normal breast tissues (NORMA1-4). For example, on gene expression analysis there were very low significant levels of all ADSC stem cell markers in ADSC of TRIDUC1, and only 0.5-fold to 1.0-fold of markers in IFDUC1, when compared to the mean of NORMA1-4 (Fig. 5a, Additional file 2e, f). Interestingly, the stem cell marker *CD36* was expressed approximately 7.0-fold higher in both the ADSC NORMA1-4 and the ADSC IFDUC1, when compared to ADSC TRIDUC1 cells (Fig. 5a, Additional file 2e, f). Considering mesenchymal or muscle specific marker genes, there was generally greater expression for both tumor MES primary cell lines when compared to NORMA1-4 MES cell lines (Fig. 5b, Additional files 2, 3 and 4). For example, *VIM* expression in MES IFDUC1 and TRIDUC1 cells demonstrated 8.0-fold to 12.0-fold higher levels, when compared to MES cells of NORMA1-4. Significantly higher levels of the *CDH2* and *CDH11* mesenchymal genes were observed, especially for IFDUC1 MES cells compared to NORMA1-4 primary breast cell lines (Fig. 5b).

Both EMT marker genes, *SNAI1* and *ARHGAP22*, were expressed 17.4-fold and 12.4-fold higher in IFDUC1 MES than NORMA1-4 MES, respectively, suggesting an active invasive state (Fig. 5b). Further support for activated invasion in tumor MES was indicated with *MMP2* gene expression, which was over 8.5-fold higher for MES of IFDUC1 compared to NORMA1-4 MES (Fig. 5b).

On comparing the tumor MEC primary cell line from IFDUC1 with all NORMA1-4 cell lines, only minor differences could be detected in general. While *KRT18/19, MUC1* and *CNN1* were expressed to a lower extent in MEC IFDUC1, there was significantly greater expression of *CD34, CD45, CD24, CD49f, CDH1 CDH3* and *KRT5* when compared to NORMA1-4 MEC primary cell lines (Fig. 5c, d, Additional file 4e).

Last, we analyzed the expression of novel breast cancer targets *MCAM/CD146* and *SOD1* in the cell lines [32, 33]. Interestingly, there was significantly greater expression of *MCAM/CD146* in MEC IFDUC1 compared to MEC NORMA1-4. Comparing MES NORMA1-4 with MES TRIDUC1 we detected a significant increase in expression of *SOD1* in cancer cells (Fig. 5e).

### Culture of isolated ADSC resulted in differentiation of four separate cell lineages

To test the multipotent nature of ADSC we performed a variety of cell culture studies following fractionation. Initially, after isolation and seeding we observed that the cells were round-shaped and then became fully attached within 24 h. In approximately 1 week of culturing the cells grew exponentially and had a typical and homogenous mesenchymal-like appearance (Fig. 1a).

**Fig. 6:**
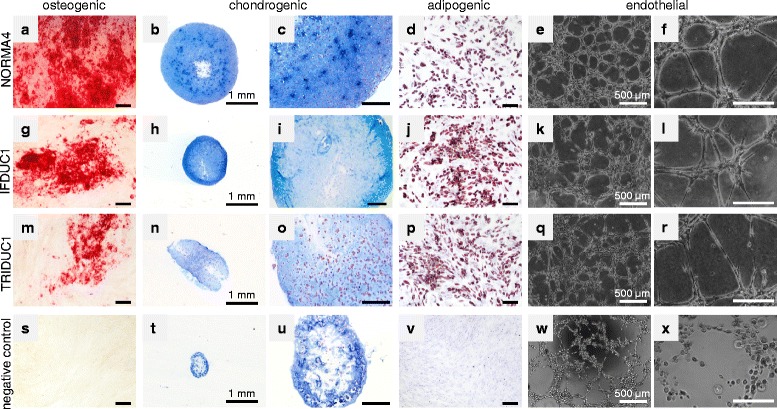
Multipotent adipose-derived stem cell (ADSC) differentiation into four different cell lineages. For verifying the multipotency of isolated ADSC from normal and tumor breast tissue, cells were differentiated into the osteogenic, chondrogenic, adipogenic and endothelial cell lineages. Osteogenic differentiation was demonstrated by Alizarin red staining (**a**, **g**, **m**), chondrogenic differentiation by Alcian blue staining (**b**, **c**, **h**, **i**, **n**, **o, t**, **u**), adipogenic differentiation by Oil Red O staining (**d**, **j**, **p**) and endothelial-like capillary tube structures (**e**, **f**, **k**, **l**, **q**, **r**). Negative control groups cultivated in standard cell culture medium, but without additional supplements, showed no positive staining (**s**-**v**). Normal (*NORMA*)4 mammary epithelial cells represented a negative control for endothelial-like tube assays (**w**, **x**). *Bar* = 200 μm. *IFDUC* invasive inflammatory ductal carcinoma, *TRIDUC* triple-negative ductal carcinoma

To verify the multipotency of ADSC we performed functional analyses, where normal and tumor ADSC were differentiated into osteogenic, chondrogenic, adipogenic, and endothelial-like lineages (Fig. 6). NORMA1-4 ADSC differentiated similarly into all four cell lineages thus, were comparable (Fig. 6a-r and data not shown). As an example, NORMA4 had strong osteogenic differentiation as demonstrated with Alizarin red staining detecting extracellular calcium deposits (Fig. 6a). Alcian blue staining revealed the presence of glycosaminoglycan after chondrogenic differentiation (Fig. 6b, c). Adipogenic differentiation was shown by staining of lipid droplets with Oil Red O (Fig. 6d). Last, negative control groups for all of the above were cultivated in standard culture medium alone and there was no positive staining for all lineages (Fig. 6s-v).

It is well-known that endothelial cells form unique branching capillary-like structures on Matrigel^®^, where one hallmark is the presence of single mesenchymal spindle cells that stretch and line the capillary lumen [25, 34]. Interestingly, within 5 h after the seeding of normal breast ADSC onto polymerized Matrigel^®^ beds, endothelial-like capillary structures were noted, thus supporting that there was differentiation towards angiogenesis (Fig. 6e, f). Although normal control breast MEC formed web-like colonies (Fig. 6w, x), only ADSC demonstrated capillary structures consisting of single mesenchymal-like cells bordering the capillary lumen (Fig. 6f). Therefore, normal breast MEC cells demonstrated cell-cell adherence properties but represented a negative control in endothelial-like characteristics. Taken together, primary ADSC from normal breast tissues differentiated in culture into four separate cellular lineages and thus, retained their stem cell nature after isolation.

Additionally, we performed differentiation studies comparing NORMA4 with ADSC isolated from IFDUC1 and TRIDUC1. Osteogenic differentiation was impaired for both tumor cell lines compared to NORMA4 (56.9 % for IFDUC1 and 36.1 % for TRIDUC1 compared to NORMA4 set to 100 %) (Fig. 6a, g, m). Chondrogenic differentiation was also reduced for both tumor ADSC (NORMA4 was 12.85-fold higher than negative control set to 1; IFDUC1 was 4.42-fold and TRIDUC1 4.38-fold higher than the negative control) (Fig. 6b, c, h, i, n, o). On the other hand, for adipogenic differentiation of tumor, ADSC was higher compared to NORMA4 set to 1 (IFDUC1 1.52-fold; TRIDUC1 1.38-fold) (Fig. 6d, j, p). We interpret our findings that the tumor environment is negatively influencing tumor ADSC to differentiate into osteogenic and chondrogenic lineages but positively influencing tumor ADSC towards adipocyte differentiation.

Interestingly, both breast cancer ADSC also differentiated into classical endothelial-like capillary structures similar to normal breast ADSC (Fig. 6e, f, k, l, q, r). However, on measuring different endothelial-like structural characteristics there were significant differences between tube length and area when comparing breast cancer and normal ADSC (Additional file 5a-c). Tubes were significantly longer in TRIDUC1 compared to NORMA4, suggesting increased cellular physical strength. Although the total capillary loop formation did not differ significantly between breast cancer and normal ADSC, the endothelial-like area of complete and non-complete capillary structures was significantly smaller for both TRIDUC1 and IFDUC1 when compared to NORMA4, indicating more complete endothelial-like differentiation for the tumor ADSCs.

### Conditioned media from tumor and normal ADSC differentiating into adipocytes induced an EMT with increased 3D invasion of breast cancer MEC

**Fig. 7:**
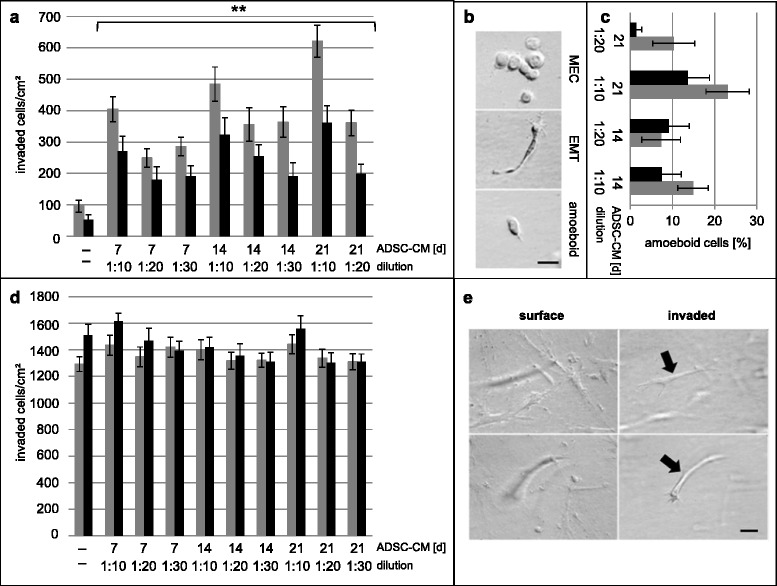
Three-dimensional collagen invasion assays and quantification of cell phenotypes of invasive inflammatory ductal carcinoma (IFDUC)1 mammary epithelial cells (*MEC*) and mesenchymal cells (MES) incubated with conditioned medium (CM) from different adipose-derived stem cell (*ADSC*)-differentiated adipocyte stages. IFDUC1-MEC and IFDUC1-MES cells were incubated with CM from ADSC-IFDUC1 and ADSC-normal mammary cells (NORMA)4 during adipocyte differentiation at the time points 7, 14 and 21 days to test the influence on invasion for 72 h. *Y*-*axis* represents total number of invaded IFDUC1-MEC cells (invaded cells/cm^2^); *X-axis* represents ADSC-CM for control (−− = no CM), and CM at 7, 14 and 21 days; *gray bar* represents CM from ADSC-IFDUC1 and *black bar* from ADSC-NORMA4. Directly *below* three CM dilutions (1:10, 1:20 and 1:30) were tested and correspond with specific days (**a**). Examples of IFDUC1-MEC cells on the collagen surface (*top*) and invaded mesenchymal or amoeboid phenotypes below the collagen surface (*middle* and *bottom*), supporting epithelial to mesenchymal transition (*EMT*) or epithelial to amoeboid transition; *bar* = 50 μm (**b**). *Y-axis* represents total number of invaded IFDUC1-MES cells (invaded cells/cm^2^); *X-axis* represents ADSC-CM for control (−− = no CM), and CM at 7, 14 and 21 days; *gray bar* represents CM from ADSC-IFDUC1 and black bar from ADSC-NORMA4. *Directly below* three CM dilutions (1:10, 1:20 and 1:30) were tested and correspond with specific days (**c**, **d**). IFDUC1-MES cells on the collagen surface (*left*) and from the same field invaded cells below the collagen surface; invaded cells are out of focus (*left* panels) but become clear when focusing below the surface (*right* panels). *Bar* = 50 μm (**e**)

We designed cell culture studies to address a possible tumor function for the overproduction of breast adipocytes derived from tumor ADSC compared to normal breast ADSC (Fig. 6 d, j, p; Fig. 7). Our approach involved isolating CM from ADSC-IFDUC1 and ADSC-NORMA4 during adipocyte differentiation at three time points over 21 days. IFDUC1-MEC and IFDUC1-MES were then incubated with the above CM to test the influence on invasion using 3D collagen matrices for 72 h. CM from all adipocyte differentiation stages significantly increased the invasion of IFDUC1-MEC, where CM at week 3 induced the greatest invasion (Fig. 7a). Additionally, IFDUC1-MEC invasion was augmented with CM from differentiated IFDUC1-ADSC compared to NORMA4-ADSC (Fig. 7a). Thus, CM from both breast tumor and normal differentiating ADSC significantly induced invasion of tumor MEC cells, supporting a role of critical adipokine factors. Importantly, all collagen-invaded IFDUC1-MEC consisted mainly of a mesenchymal phenotype and fewer amoeboid phenotypes, supporting that there is enhancement of an EMT or an epithelial to amoeboid transition (Fig. 7b, c). In contrast, IFDUC1-MES cells showed no significant increase of invasion in the presence of CM, but demonstrated high numbers of invaded mesenchymal cells, indicating innate high invasiveness of breast tumor MES (Fig. 7d, e).

### Multiple PCL fiber scaffolds support adhesion and initiation of cellular growth

**Fig. 8:**
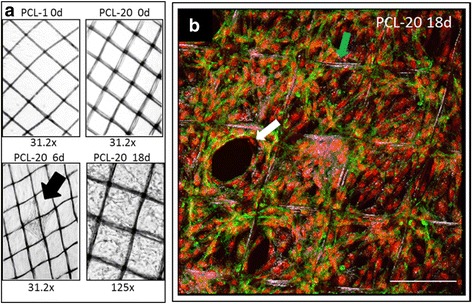
Three-dimensional culture of breast mesenchymal cells for tissue engineering purposes. **a** Phase contrast microscope image shows the two melt electrospinning writing poly(ε-caprolactone) (*PCL*) scaffolds, *PCL-1* (one layer of fibers) and *PCL-20* (20 layers of fibers) at cell seeding of the normal (NORMA)2 mesenchymal cell (MES) cell line and then consecutively at 6 and 18 days of growth. Note that no growth occurred on PCL-1 throughout the 18-day period. **b** Confocal microscope image shows NORMA2 MES after 18 days of growth. *Image* represents an overlay of 43 z-stack sections (each step 1.43 μm) equaling 61.49 μm. *Green* represents the WGA Alexa 488 fluorescent cellular membrane and the *red* the DRAQ5 (633 nm)-stained nuclei. *Blue arrow* indicates the PCL scaffold overlaid with the bright field channel. *White arrow* indicates an area with ongoing cell growth, which originally initiated along the fibers. *Bar* = 200 μm

Presently, there is a need to study the growth of primary cells on biomaterials applicable for tissue regeneration, for example, breast reconstruction. We therefore studied the growth properties of primary normal breast cells on fine, 3D-printed PCL scaffold substrates (Fig. 8a, b) with fiber diameters of 14 ± 1 μm. A 2.5D scaffold consisting of only a single strand layer (PCL-1) was compared to a 3D scaffold with 20 alternating fiber layers (PCL-20) for growth with primary NORMA2 MES cells. Following the seeding of MES cells, we observed that cellular growth initiated from the fiber frame within 6 days. Interestingly, these cells appeared to represent a bridge facilitating further proliferation toward the scaffold center. After 18 days increased cellular confluency of >90 % was observed throughout the 3D scaffold with PCL-20 (Fig. 8a, b), but in contrast no cell growth occurred with PCL-1 (Fig. 8a). Our results indicate how cell adhesion and proliferation is affected through the use of 3D scaffold structures.

## Discussion

In 1958 an epithelial cell line called BT-20/HTB-19 was established directly from a solid invasive ductal breast carcinoma [35]. Introduced later were many other breast cancer cell lines [4], of which two of the best known are MCF-7 (HTB-22) and MDA-MB231 (HTB-26). However, most of these cell lines, including MCF-7 and MDA-MB231, did not directly originate from the primary tumor (Table 2). MCF-7 was generated from a pleural effusion more than 3 years after mastectomy and radiation/hormone therapy; MDA-MB231 was also initiated from a pleural effusion 4 years after mastectomy [36, 37].

**Table 2:** A short overview of the most common breast cancer cell lines and their origin

Cell line (ATCC)	Origin	Carcinoma/normal	Clinic-pathology
MCF-7 (HTB-22)	pleural effusion	adenocarcinoma	luminal A
T47D (HTB-133)	pleural effusion	ductal carcinoma	luminal A (ER+, Her2–)
MDA-MB-134 (HTB-23)	pleural effusion	ductal carcinoma	epithelial
MDA-MB-231 (HTB-26)	pleural effusion	adenocarcinoma	Claudin low (ER–)
MDA-MB-436 (HTB-130)	pleural effusion	adenocarcinoma	pleomorphic and multinucleated
MDA-MB-453 (HTB-131)	pericardial effusion	metastatic carcinoma	epithelial
MDA-MB-468 (HTB-132)	pleural effusion	adenocarcinoma	basal (ER–, EGFR++)
CAMA-1 (HTB-21)	pleural effusion	adenocarcinoma	epithelial
SK-BR-3 (HTB-30)	pleural effusion	adenocarcinoma	Her2+ (ER–)
HCC1428 (CRL-2327)	pleural effusion	adenocarcinoma	epithelial (ER+, Her2–)
SUM149PT	breast	inflammatory carcinoma	basal B (ER-, PR–)
SUM190PT	breast	inflammatory carcinoma	luminal (ER–, PR–, Her2+)
BT-20 (HTB-19)	breast	ductal carcinoma	epithelial (ER–, EGFR++)
BT-157 (HTB-122)	breast	ductal carcinoma	mesenchymal (ER–)
BT-474 (HTB-20)	breast ducts	ductal carcinoma	luminal B (ER+, Her2+)
Hs578T (HTB-126)	breast	carcinoma	luminal A (ER–)
HCC1954 (CRL-2338)	breast ducts	ductal carcinoma	epithelial (ER-, HER2++)
HCC1143 (CRL-2321)	breast ducts	ductal carcinoma	epithelial (ER–)
MCF10A (CRL-10317)	breast	normal	epithelial adherent
MCF10F (CRL-10318)	breast (see MCF10A)	normal	epithelial floating
hTERT-HME1 (CRL-4010)	breast	normal	epithelial

The origin, the clinic-pathology and the receptor status are from [3, 68, 69] and information from ATCC (www.atcc.org/). *ER* estrogen receptor, *Her2* human epidermal growth factor-2

In addition to the question of cell comparability between primary tumors and pleural effusions, and the reproducibility of tumor-cell-related gene/protein and signaling, many cell lines are contaminated; e.g., human cervix carcinoma HeLa cells with rodent cells or other misidentified cells [38, 39]. Therefore, it is essential to implement tests to genomically verify the identity of cell lines [39] like DNA fingerprinting, which we demonstrated for one patient’s primary tissue and isolated cell lines.

Based upon several publications four major cell types within normal human breast tissue have been assigned using both gene and protein expression. This profile is the following: 1) luminal 1 epithelial cells (*EPCAM* high/*CD49f*-/*CD24*+/*KRT8*, *18* high/*KRT14*-/*ACTA1*- (alpha skeletal muscle actin)/*VIM* low); 2) luminal 2 epithelial cells (*EPCAM* high/*CD49f*+/*CD24*+/*KRT8*, *18* high/*KRT14*-/*ACTA1*-/*VIM*+); 3) basal epithelial cells (*EPCAM* low/*CD49f*+/*CD24*-/*KRT8*, *18* low/*KRT14*+/*ACTA1* high/*VIM* high) and 4) mesenchymal cells (*EPCAM*-/*CD49f*+/*CD24*-/*KRT8*, *18* low/*KRT14* low/*ACTA1* low/*VIM* high) [9, 17, 40]. However, comparing primary normal breast tissues (*n* = 12) with established normal mammary epithelial cell lines, like HME I (CRL-4010) and HME II, MCF10A (CRL-10317) and 10 F (CRL-10318), Keller et al. conclude that these particular cell lines do not retain the cellular diversity found in human breast tissues, but appear to be enriched for differentiation states that represent a minority of breast tissues [9]. These findings suggest that only assemblies of cell lines representing multiple cell types can be used to model the cellular heterogeneity of tissues. Considering the most well-known established normal and tumor breast cell lines for research studies, almost all cell lines resulted in only one of the existing cell type lineages (Table 3).

**Table 3:** Primary cells confirm the heterogeneity of the tissue

	Cell types	*EPCAM*	*CD49f*	*CD24*	*MUC1*	*KRT8*	*KRT18*	*KRT14*	*ACAT1*	*VIM*
Gene expression signature according to [9, 17, 40]	luminal 1	high	--	+	+	high	high	--	--	low
	luminal 2	high	+	+	+	high	high	--	--	+
	basal-like	low	+	--	--	low	low	+	high	high
	mesenchymal	--	+	--	--	low	low	low	low	high
	cell lines									
Gene expression of IFDUC1 cell lines compared to of NORMA1-4	MEC of IFDUC1	low 2.8 × lower	+ 1.4 × higher	+ 2 × higher	--	low 1.8 × lower	low 2.6 × lower	+ equal	high 2.5 × higher	high 1.3 × higher
	MES of IFDUC1	+ 1.7 × higher	low 1.5 × lower	--	high 7.8 × higher	low 5.8 × lower	+	--	high 7.7 × higher	high 10 × higher
	ADSC of IFDUC1	--	low 1.8 × lower	--	low 1.8 × lower	low 1.5 × lower	+ equal	--	high 1.6 × higher	high 1.3 × higher

For isolated primary cells the lower/higher values correspond to the mean of normal mammary cells (NORMA)1-4 for mammary epithelial cells (*MEC*), mesenchymal cells (MES) or adipose-derived stem cells (*ADSC*). Fold-changes (×) represent lower, equal or higher expression of invasive inflammatory ductal carcinoma (*IFDUC*)1 cell lines compared to NORMA1-4 mean values

The conclusion of heterogeneity of normal breast tissues or tumors between individuals (heterologous) and a patient’s normal tissue and the tumor itself (autologous) led us to perform simultaneous isolations of MEC, MES and ADSC from normal breast and tumor tissues from the same individual. Therefore, our study presented the opportunity to combine gene expression and protein analyses from isolated breast cell types and perform a direct comparison with published gene expression profiles of normal and tumor cell lineages. In addition, these cell types can easily be implemented into specific research applications, such as cell functional or drug assays. For example, our functional studies demonstrating that PCL-20 3D-scaffolds containing 20 fibers (but not single-strand (or 2.5D) scaffolds [41]) supported adhesion and growth of normal breast MES, have important future clinical implications, especially for breast cancer patients. Although, more studies are needed one could envision that using such 3D-printed scaffolds for re-growing normal breast tissue ex vivo for possible later implantation, has translation applications for the expanding field of regenerative medicine [14, 42–44].

Comparing the four published normal breast cell lineages (luminal (1 and 2) epithelial, basal-like epithelial and mesenchymal cells) gene/protein expression levels with our normal and breast tumor cell lines assisted us further to confirm cell lineages (Table 3). Interestingly, we interpret that NORMA1 and NORMA2 MEC primary cell lines resembled luminal 1 epithelial cells, whereas NORMA3 and NORMA4 resembled luminal 2 epithelial cells. Additionally, we predict that the IFDUC1 MEC line has a very high conformity of gene expression (*EPCAM*, *CD49f*, *MUC1*, *KRT8*/*18*/*14*, *ACTA1* and *VIM)* to the basal-like epithelial cell lineage (Table 3). However, expression of *CD24*, which is normally expressed in luminal epithelial cells, was 2-fold higher in the IFDUC1 MEC line, thus, was in contrast [9]. Other researchers also showed that breast tumors express *CD44* and *CD24* but are very heterogeneous among different tumors and also within the tumor [45]. The latter analysis also found a *CD44*+/*CD24*- phenotype as the most common in the basal-like subgroup with *ER*-/*PR*-/*Her2*-/*KRT5*/*14*+ and in BRCA1 hereditary tumors. MEC cells of IFDUC1, derived from a tumor characterized as *ER*+/*PR*+/*Her2*+ and non-BRCA1 hereditary were both *CD44*+ and *CD24*+. In addition, *KRT19* expression representing luminal cells was negative for MEC of IFDUC1, whereas *KRT5* expression representing basal-like cells was positive in MEC of IFDUC1. On the other hand, *CD10* expression, representing a basal, bi-potent and myoepithelial cell phenotype was also highly expressed in MEC of IFDUC1. Thus, the *KRT14*+, *CD49* high, *CD10* high and *MUC1* expression supports the presence of a high portion of bi-potent progenitors in the IFDUC1 MEC primary cell line. Additionally, our findings of *CD34+/CD45+* IFDUC1 MEC further support more undifferentiated tumor cells [46].

Analyzing IFDUC1 MES and ADSC primary cell lines with the expression signature of the four main cell lineages, especially the mesenchymal lineage, we found partial similarities with *CD49f*, *CD24, KRT8, 14* and *18* and *VIM* (Table 3). Importantly, the elevated levels of *VIM* in IFDUC1 and TRIDUC1 MES and ADSC primary cell lines supports an invasive potential [47], but could also reflect breast progenitor cells with a bilinear (glandular and myoepithelial) differentiation potential [48]. Our finding that IFDUC1 MES cells additionally expressed significantly higher levels of *SNAI1* and *MMP2* along with a high invasion capability (mean of 1400 invaded cells per cm^2^ in 3D collagen matrices) further corroborates a high invasive phenotype for MES cells isolated from a patient with an inflammatory breast tumor. Taken together, the three isolated cell lineages MEC, MES and ADSC from IFDUC1 reflect the heterogeneity normally found in tumors and confirm the need to establish different cell lines from one tissue to represent its entirety.

There are approximately 0.6 × 10^6^ ADSC per gram of breast tissue total, and nearly 6 % of these cells are expected to be adherent and highly proliferative [49]. It is well-known that these cells cannot be characterized on the basis of one single cell surface marker and therefore a large variety of positive and negative markers are used for the characterization of ADSC in different studies (reviewed in [50]). In 2006 the International Society for Cellular Therapy (ISCT) developed the minimal criteria for defining multipotent mesenchymal stromal cells [51]: (a) adherence to plastic, (b) expression of *CD105*, *CD73*, *CD90*, absence of *CD45*, *CD34*, *CD14* or *CD11b*, *CD79α* or *CD19* and *HLA-DR*, and (c) multipotent differentiation potential (adipogenic, chondrogenic, osteogenic). In the present study, isolated ADSC from NORMA1-4 fulfill these criteria, where we observed high expression of *CD105*, *CD73* and *CD90* in ADSC compared to the other primary cell types and the absence of *CD14*, *CD19*, *CD45* and *HLA-DR*. However, *CD34* expression was generally greater but did differ among the patients. While the criteria of the ISCT were reported in a range of publications to have been applied for the characterization of ADSC, others doubt the applicability, because of the expression of *CD34* in early passages of ADSC [52]. In an analysis by Baer et al. in ADSC from 16 patients there was high variability in the expression profile of *CD36* and *CD34*, probably due to the age, body mass index, ethnicity or medical history of the donors [53]. While some markers increase rapidly after plating, such as *CD29*, *CD44*, *CD49d*, *CD73*, *CD90*, *CD105* and *CD151*, others, such as *CD31* and *CD45*, decrease. The adipocyte marker *CD36* is often used for distinguishing mesenchymal stem cells from bone marrow and adipose tissue; we observed high expression in all four of our normal and in one breast tumor ADSC primary cell lines [54]. Furthermore, we found that the stem cell markers *CD29* and *CD44* [55–57] were expressed in NORMA1 ADSC MEC and MES but not significantly differently. Only some ADSC will lose *CD34* expression with increasing culture time, while others maintain it for at least 10–20 weeks [57]. Suga et al. suggested that loss of *CD34* expression can be seen as a physiological process of maturation and thus, correlates with replicative capacity or the differentiation potential of ADSC [58]. In this present study we support the idea that the significant higher expression of known endothelial markers *CD34* and *PDGFRB* noted on both normal and tumor ADSC could be linked with the potential of ADSC to differentiate into an endothelial-like lineage, as discussed below. Furthermore, the highest expression of *PDGFRB* noted in ADSC TRIDUC1, could verify the overall worse prognosis of this cancer type, due to the finding that high stromal *PDGFRB* expression is associated with poor prognosis among patients with breast cancer [59].

Our findings that both IFDUC1 and TRIDUC1 ADSC lines had lower levels of the marker genes (*CD105*, *CD73*, *CD90*, *CD44*, *CD106*) when compared with NORMA1-4, supports a link with impaired tumor ADSC differentiation into osteogenic and chondrogenic lineages. This suggests that the tumor environment may negatively influence the ability of the ADSC to differentiate into these two lineages. On the other hand, our findings that both IFDUC1 and TRIDUC1 ADSC differentiated into significantly more complete endothelial-like capillary tube structures as compared with NORMA1-4 demonstrates that cultured breast ADSC also have an angiogenesis potential, similar to freshly isolated and non-adherent ADSC from non-breast subcutaneous liposuction, which differentiate in vitro into vascular endothelial cells, vascular smooth muscle cells and cardiomyocytes [60]. Thus, breast tumor ADSC play a role in vascularization within the tumor environment.

It has been noted that normal breast ADSC can be influenced by tumor factors [61, 62]. For example, breast ADSC differentiates into a myofibroblastic phenotype following incubation with CM or with isolated exosomes from breast tumor cell lines, implicating the transforming growth factor beta signaling pathway [61, 62]. Recent findings showed that tumor-surrounding adipose tissue has a major impact on breast cancer progression, following activation of the adipocytes (so-called cancer-associated adipocytes) through tumor cells [63]. For example, local tumor cell invasion occurs early and is closely associated with white adipose tissue consisting of progenitor and mature adipocytes [64]. Co-culture systems using heterologous primary human ADSC (from lipo-aspiration and breast) with immortalized breast tumor cell lines have demonstrated increased tumor proliferation, migration, invasion, EMT and secretion of different factors, e.g., inflammatory cytokines (IL-6, IL-8) and matrix metalloproteinases (MMPs) [65, 66]. Our autologous and heterologous co-cultures involving primary tumor ADSC, MEC and MES (IFDUC1) and NORMA4 ADSC implies important aspects of the breast tumor environment. Tumor-associated ADSC exhibit increased plasticity towards adipocyte differentiation, which has a direct significant effect, inducing 3D invasion of tumor MEC via an EMT and amoeboid cells. Our data support the idea that tumor MEC invasion was induced by CM-adipokine factors from both early and mature adipocytes, but interestingly, had no effect on tumor MES. Furthermore, IFDUC1-positive CD34 ADSC could be a contributing metastatic factor as CD34+ ADSC were shown to induce a metastatic shift of breast cancer cell lines in a mouse model [67]. Last, CM-adipokines from NORMA4 ADSC differentiating into adipocytes induced invasion of tumor MEC, but to a lower degree than tumor ADSC. These findings further point to a possible negative impact of ADSC on residual breast cancer cells in patients who receive AFT for esthetic purposes.

## Conclusion

In the present study we successfully isolated three different cell phenotypes from human primary normal and tumor breast tissue. We could establish a molecular signature for MEC, MES and ADSC and propose that only the ensemble of all cells can represent the tissue or tumor adequately. As commonly used breast cell lines only represent specific cell types or differentiation states, we propose to use assemblies of different cell lines from the same patient.

Tumor-associated ADSC have an impaired plasticity towards chondrogenic and osteogenic differentiation, but an increased adipogenic and endothelial-like differentiation, supporting the idea of a stimulus on invasion and angiogenesis in the tumor environment. Co-culturing of isolated autologous and heterologous primary cell lines could help us to understand gene expression, differentiation, invasion, hormone and inhibitor/drug interactions of control versus tumor breast tissue in vitro that are important for tumor biology and regenerative medicine.

## Additional files

Additional file 1: Primer sequences. (DOCX 18 kb) Additional file 2: Gene expression of stem cell and hematopoietic markers in cell isolates. Mammary epithelial cell (*MEC*), adipose-derived stem cell (*ADSC*) and mesenchymal cell (*MES*) primary cell lines all from the same patients (four normal (*NORMA1-4*) and two breast tumor primary cell lines are indicated). **a**-**f** Expression results of positive stem cell markers *CD105, CD73, CD90, CD36, CD29, CD44* and *vimentin (VIM)* are shown in four NORMA1-4 and two breast tumor (invasive inflammatory ductal carcinoma (*IFDUC*)1, triple-negative ductal carcinoma (*TRIDUC*)1) different primary cell lines using real-time PCR (**p* ≤0.05). **g**-**l** Hematopoietic markers, *CD14, CD19, CD34, CD45* and *HLA-DR* are shown from peripheral blood cells (PBC)/blood endothelial cells (*BEC*), four NORMA1-4 and two breast tumor (IFDUC1, TRIDUC1) primary cell lines using real-time PCR (**p* ≤0.05). (PPTX 135 kb) Additional file 3: Gene expression of invasive and mesenchymal markers in cell isolates. Mammary epithelial cell (*MEC*), adipose-derived stem cell (*ADSC*) and mesenchymal cell (*MES*) primary cell lines all from the same patients (four normal (*NORMA1-4*) and two breast tumor primary cell lines (invasive inflammatory ductal carcinoma (*IFDUC*)1, triple-negative ductal carcinoma (*TRIDUC*)1)) are indicated. Expression results using real-time PCR of genes involved in invasion, *MMP2, MMP9* and *SNAI1* and markers of the mesenchymal phenotype, *N-cadherin (CDH2)* and *cadherin11 (CDH11)* (**p* ≤0.05). (PPTX 114 kb) Additional file 4: Gene expression of typical epithelial cell markers in cell isolates. Mammary epithelial cell (*MEC*), adipose-derived stem cell (*ADSC*) and mesenchymal cell (*MES*) primary cell lines all from the same patients (four normal (*NORMA1-4*) and the invasive inflammatory ductal carcinoma (*IFDUC*)1 breast tumor primary cell line) are indicated. Expression results are shown for a range of typical markers for characterization of epithelial cells, the proliferation marker *MKI67* and markers of basal myoepithelial mammary cells (*ACTA1*, *ACTA2*, *CNN1*, *CD10*) using real-time PCR (**p* ≤0.05). (PPTX 132 kb) Additional file 5: Endothelial-like differentiation of adipose-derived stem cells (ADSC) into capillary structures. Endothelial-like differentiation was quantified 5 h after seeding ADSC on Matrigel®. **a** *X axis* represents the tube length in mm for each capillary side cell structure. **b** *X axis* represents the number of completely closed capillary loop structures. **c** *X axis* represents the area in mm^2^ for completely closed and open capillary structures. **a**-**c** *Y axi*s represents the origin of the ADSC (triple-negative ductal carcinoma (*TRIDUC*)1, invasive inflammatory ductal carcinoma (*IFDUC*)1 or normal breast (*NORMA*)4). Quantification was performed in three wells (total area 0.125 cm^2^/well). (PPTX 52 kb)

## Abbreviations

ACTA1: alpha skeletal muscle actin
ACTA2: alpha smooth muscle actin
ACVRL1: activin receptor-like kinase 1
ADSC: adipose-derived stem cells
AFT: autologous fat transfer
ARHGAP22: Rho GTPase activating protein 22
BEC: blood endothelial cells
CDH1: E-cadherin
CDH2: N-cadherin
CDH3: P-cadherin
CDH5: VE-cadherin
CM: conditioned media
CNN1: calponin
DAPI: 4',6-diamidino-2-phenylindole
DMEM: Dulbecco’s modified Eagle’s medium
ECM: extracellular matrix
EDTA: ethylenediaminetetraacetic acid
EGF: epidermal growth factor
EMA/MUC1: epithelial membrane antigen
EMT: epithelial to mesenchymal transition
EPCAM: epithelial cell adhesion molecule ER estrogen receptor
FCS: fetal calf serum
FLT1: Fms-related tyrosine kinase 1
FLT4: Fms-related tyrosine kinase 4
Her2: human epidermal growth factor receptor-2
IFDUC: invasive inflammatory ductal carcinoma breast cancer
IL: interleukin
KDR: kinase insert domain receptor
KRT: cytokeratin
MCAM: melanoma cell adhesion molecule/CD146
MEC: mammary epithelial cells
MEM: minimum essential medium
MES: mesenchymal cells
MEW: melt electrospinning writing
MMP: matrix metalloprotease
NORMA: normal mammary cells
P: pellet
PBC: peripheral blood cells
PBS: phosphate-buffered saline
PCL: poly(ε-caprolactone)
PDMS: polydimethylsiloxane
PECAM1: platelet endothelial cell adhesion molecule
PR: progesterone receptor
RBC: red blood cells
RT: room temperature
S: supernatant
STR: short tandem repeats
TRIDUC: triple-negative ductal carcinoma VEGF, vascular endothelial growth factor
VIM: vimentin
vWF: von Willebrand factor
YWHAZ: tyrosine 3-monooxygenase/tryptophan 5-monooxygenase activation protein, zeta
