# Supplementary material for: Selective isolation and characterization of primary cells from normal breast and tumors reveal plasticity of adipose derived stem cells
Source: Breast Cancer Res. 2016 Mar 12;18:32. doi: 10.1186/s13058-016-0688-2 (PMC4788819; doi:10.1186/s13058-016-0688-2)
Supplement: Additional file 1: — Primer sequences. (DOCX 18 kb) [file 13058_2016_688_MOESM1_ESM.docx]

| **gene** | **Forward 5‘-3‘** | **Reverse 5‘-3‘** |
| --- | --- | --- |
| *ACTA1* | GAGAGGTATCCTGACCCTGAAGT | AGCTCGTTGTAGAAGGTGTGGT |
| *ACTA2* | ACTGCCTTGGTGTGTGACAA | CGTCCCACAATGGATGGGAA |
| *ACVRL1* | CTTTGAGTCCTACAAGTGGACTGA | TAGAAGGGTGGTCTATAGTCCTCC |
| *ARHGAP22* | GGAGTTGGCTAAACAAGTGAGC | GCCTGAACTTCATCCAGAAACT |
| *CD10* | GATAAGTGGAGCAGCTGTAGTCAA | CATAGTTCAATGAGTTGGACTGCT |
| *CD105* | CCAAGACCGGGTCTCAAGAC | TGTACCAGAGTGCAGCAGTG |
| *CD106* | TGTCATGGGCTGTGAATCCC | CTCAGGGTCAGCGTGGAATT |
| *CD14* | ACAGGTGCCTAAAGGACTGC | AGTTCATTGAGCCCTCGTGG |
| *CD19* | TGCCCCGTCTTATGGAAACC | TAGCCCTCCCCTTCCTCTTC |
| *CD24* | CCAGTGAAACAACAACTGGAAC | TGCAGAAGAGAGAGTGAGACCA |
| *CD29* | GTCGTGTGTGTGAGTGCAAC | TTGCAGATCTGTCCGTTGCT |
| *CD34* | CAGCAAGACAACACGTGGTG | CCCAAGAACAGCCTCTGAGG |
| *CD36* | CGCTGAGGACAACACAGTCT | CTGCCACAGCCAGATTGAGA |
| *CD44* | CTCCAGTGAAAGGAGCAGCA | GCAGGGATTCTGTCTGTGCT |
| *CD45* | TCAGTGGTCCCATTGTGGTG | GCATCTCTGTCGCCTTAGCT |
| *CD49f* | AGGATGGGTGGCAAGATATAGTT | CATTAAGACGAATTGGCTTCACATT |
| *CD73* | TATCCGGTCGCCCATTGATG | ACGCTATGCTCAAAGGCCTT |
| *CD90* | ATCAGGAGTTCCAGTGCTGC | TGGCTTCCCTCTTCACGAAC |
| *CDH1* | TCATGAGTGTCCCCCGGTAT | TCTTGAAGCGATTGCCCCAT |
| *CDH11* | AATTAGTGCAGATGACAAGGATGA | TGTCTCTGACTGTGAAATTTGGAT |
| *CDH2* | TCAATGACAATCCTCCAGAGTTTA | TGATCCTTATCGGTCACAGTTAGA |
| *CDH3* | CTACCAGGTACTTCTGTGATGCAG | GTTCTTGGCTATGGATGGAGTAAG |
| *CDH5* | AATGTGGACAAGGACACTGGCGAA | ACGGACGCATTGAACAACCGAT |
| *CNN1* | TTGAGGCCAACGACCTGTTT | TTTCCGCTCCTGCTTCTCTG |
| *EPCAM* | AGCTGGCTGCCAAATGTTTG | ATCATTGTTCTGGAGGGCCC |
| *FLT1* | TAGGAAAGGGCGCCTACTCT | TTCTGACTGGCTGCAGAAGG |
| *FLT4* | TTTCCAACCCCTTCCTGGTG | AGAGCTCGTTGCCTGTGATG |
| *HLA-DR* | ACTGCAGACACAACTACGGG | ACACCACCCCAGTCTTCTCT |
| *KDR* | ACACTGGAGCCTACAAGTGC | GCCAAGTCAGTTTCCCGGTA |
| *KRT 14* | CAGTCCCAGCTCAGCATGAA | GCATGCAGTAGCGACCTTTG |
| *KRT 18* | ACAAGTACTGGTCTCAGCAGATTG | CTCCAAGGACTGGACTGTACG |
| *KRT 19* | AGGAGGAAATCAGTACGCTGAG | ATATTGGCTTCGCATGTCACT |
| *KRT 5* | TGTTCGAGCAGTACATCAACAAC | ATGTTTCTCAGCTCTGAGTCCAG |
| *KRT 7* | ACAGCTGCTGAGAATGAGTTTGT | GGAAGTTGATCTCATCATTCAGG |
| *KRT 8* | GGAAGCTGGTGTCTGAGTCC | CTGTTCCCAGTGCTACCCTG |
| *MCAM* | TGTGGGTGAAAGAGAATATGGTGT | GTGGATCTTGGTCTTGTTCACTTG |
| *MK167* | TCGACCCTACAGAGTGCTCA | GTGGGGAGCAGAGGTTCTTC |
| *MMP2* | GCCGTGTTTGCCATCTGTTT | AGCAGACACCATCACCTGTG |
| *MMP9* | CCTTGTGCTCTTCCCTGGAG | GGCCCCAGAGATTTCGACTC |
| *MUC1* | TCTGCAGGTAATGGTGGCAG | CTGGCCCTGAAGAACCTGAG |
| *PDGFRB* | TATGTCGGAGCTGAAGATCATGAG | GCGGCAGTACTCAGTGATGATATA |
| *PECAM1* | TTGACATGAAGAGCCTGCCG | CCATTTTGCACCGTCCAGTC |
| *RAC1* | TTTGACAATTATTCTGCCAATGTT | GGGCGTAATCTGTCATAATCTTCT |
| *SOD1* | TTCGAGCAGAAGGAAAGTAATGGA | AGCCTGCTGTATTATCTCCAAACT |
| *S100A4* | GCTCAACAAGTCAGAACTAAAGGAG | CTTCTGGAAAGCAGCTTCATCT |
| *SNAI1* | GCCCCACAGGACTTTGATGA | CCCTCCACAGAAATGGCCAT |
| *VIM* | AATCCAAGTTTGCTGACCTCTC | GTCTCCGGTACTCAGTGGACTC |
| *vWF* | AAGCCCATTTGCTGAGCCTTGT | AGTATCGCACAGCAAAGCCCAA |
| *YWHAZ* | ATGAGCTGGTTCAGAAGGCC | AAGATGACCTACGGGCTCCT |
