# Supplementary material for: Selective isolation and characterization of primary cells from normal breast and tumors reveal plasticity of adipose derived stem cells
Source: Breast Cancer Res. 2016 Mar 12;18:32. doi: 10.1186/s13058-016-0688-2 (PMC4788819; doi:10.1186/s13058-016-0688-2)
Supplement: Additional file 2: — Gene expression of stem cell and hematopoietic markers in cell isolates. Mammary epithelial cell (MEC), adipose-derived stem cell (ADSC) and mesenchymal cell (MES) primary cell lines all from the same patients (four normal (NORMA1-4) and two breast tumor primary cell lines are indicated). a-f Expression results of positive stem cell markers CD105, CD73, CD90, CD36, CD29, CD44 and vimentin (VIM) are shown in four NORMA1-4 and two breast tumor (invasive inflammatory ductal carcinoma (IFDUC)1, triple-negative ductal carcinoma (TRIDUC)1) different primary cell lines using real-time PCR (*p ≤0.05). g-l Hematopoietic markers, CD14, CD19, CD34, CD45 and HLA-DR are shown from peripheral blood cells (PBC)/blood endothelial cells (BEC), four NORMA1-4 and two breast tumor (IFDUC1, TRIDUC1) primary cell lines using real-time PCR (*p ≤0.05). (PPTX 135 kb) [file 13058_2016_688_MOESM2_ESM.pptx]

## Slide 1
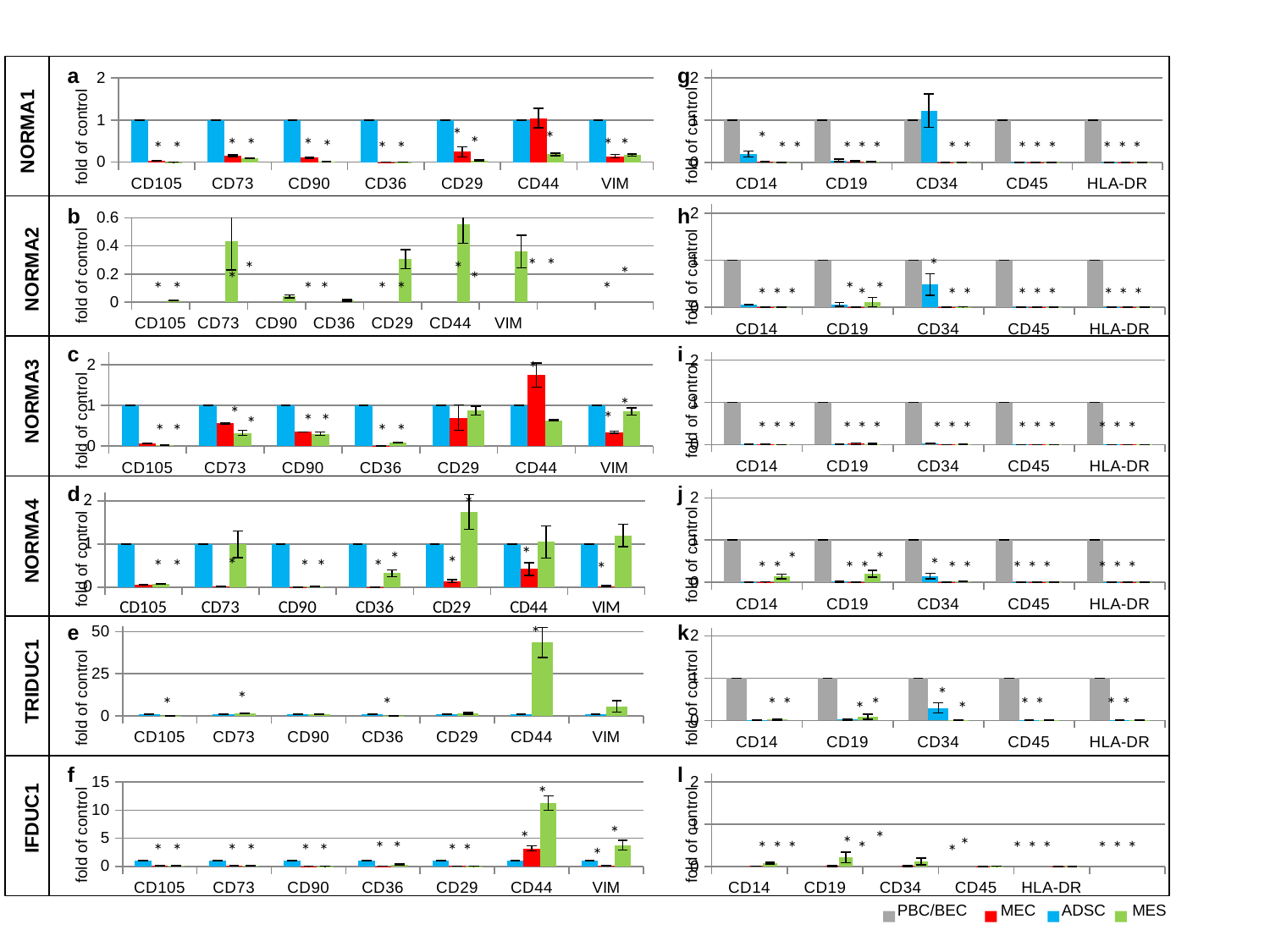

g
a
### Chart
| Category | ADSC NORMA1 | HMEC NORMA1 | MES NORMA1 |
|---|---|---|---|
| CD105 | 1.0 | 0.03181872936580483 | 0.0022039559538410513 |
| CD73 | 1.0 | 0.15182399694423696 | 0.09497197498637931 |
| CD90 | 1.0 | 0.1048065501237131 | 0.014852511072359244 |
| CD36 | 1.0 | 9.467955158524569e-05 | 0.00017145056809536835 |
| CD29 | 1.0 | 0.24438353446076433 | 0.03931976469494572 |
| CD44 | 1.0 | 1.0440916347195313 | 0.1837144043792636 |
| VIM | 1.0 | 0.1428807118740473 | 0.16763786948371054 |
### Chart
| Category | control | ADSC NORMA1 | HMEC NORMA1 | MES NORMA1 |
|---|---|---|---|---|
| CD14 | 1.0 | 0.1990416727941785 | 0.02463521373600557 | 0.001879355003731272 |
| CD19 | 1.0 | 0.04764944125010781 | 0.01937044604292647 | 0.0188720067061886 |
| CD34 | 1.0 | 1.2274437723066396 | 0.0029500114234216504 | 0.003636941228077771 |
| CD45 | 1.0 | 0.004839449894387824 | 0.00014559284593510118 | 0.0006014883465583562 |
| HLA-DR | 1.0 | 0.0003089300008046838 | 0.00010632917234946401 | 0.0010334133828878618 |NORMA1
*
*
fold of control
*
*
*
*
fold of control
*
*
*
*
*
*
*
*
*
*
*
*
*
*
*
*
*
*
*
*
*
h
b
### Chart
| Category | control | ADSC NORMA2 | HMEC NORMA2 | Mes NORMA2 |
|---|---|---|---|---|
| CD14 | 1.0 | 0.05193521467900546 | 0.0033574782011901176 | 0.0007339726976344992 |
| CD19 | 1.0 | 0.05585113859287819 | 0.0006422234381912938 | 0.10448672052385004 |
| CD34 | 1.0 | 0.4772669243066127 | 0.0023093191179932404 | 0.004722900509317359 |
| CD45 | 1.0 | 0.0035539276360104194 | 8.22809703712866e-05 | 0.0004235199562793904 |
| HLA-DR | 1.0 | 6.12549596077617e-05 | 0.0002493315256543226 | 0.002356536419416771 |
### Chart
| Category | ADSC NORMA2 | HMEC NORMA2 | Mes NORMA2 |
|---|---|---|---|
| CD105 | 1.0 | 0.02401551721035095 | 0.013812311593685607 |
| CD73 | 1.0 | 0.2699063061440539 | 0.43455292786495253 |
| CD90 | 1.0 | 0.02737592496276141 | 0.04179009609769943 |
| CD36 | 1.0 | 0.00012405671119825217 | 0.012320867964584302 |
| CD29 | 1.0 | 0.4578496193487127 | 0.3061839346422463 |
| CD44 | 1.0 | 0.5291303793175389 | 0.5565090223167023 |
| VIM | 1.0 | 0.05988641779856955 | 0.3601162289246152 |NORMA2
*
*
*
*
*
*
*
*
*
*
*
fold of control
fold of control
*
*
*
*
*
*
*
*
*
*
*
*
*
*
*
*
*
*
i
c
NORMA3
### Chart
| Category | control | ADSC NORMA3 | HMEC NORMA3 | MES NORMA3 |
|---|---|---|---|---|
| CD14 | 1.0 | 0.010712632204407428 | 0.0036618533416543906 | 0.0008744184453370545 |
| CD19 | 1.0 | 0.0045824949483202785 | 0.020498556737118145 | 0.015375033241818279 |
| CD34 | 1.0 | 0.026820476586236725 | 0.0006491219808196163 | 0.0028782410532406967 |
| CD45 | 1.0 | 0.0010515271434468614 | 1.872756714656439e-05 | 0.0004919195486284625 |
| HLA-DR | 1.0 | 8.224968361073778e-05 | 5.193807602297621e-05 | 1.5715572425822073e-05 |*
### Chart
| Category | ADSC NORMA3 | HMEC NORMA3 | MES NORMA3 |
|---|---|---|---|
| CD105 | 1.0 | 0.06023574109876049 | 0.020663433704537933 |
| CD73 | 1.0 | 0.560272109634026 | 0.3228943155054766 |
| CD90 | 1.0 | 0.34550763834570714 | 0.30024113402272823 |
| CD36 | 1.0 | 0.008673579525535562 | 0.08753096366363493 |
| CD29 | 1.0 | 0.6960164527963223 | 0.871770017760718 |
| CD44 | 1.0 | 1.7393166508670383 | 0.6290786032909828 |
| VIM | 1.0 | 0.3408263821537056 | 0.846180281623699 |*
*
fold of control
*
*
*
*
fold of control
*
*
*
*
*
*
*
*
*
*
*
*
*
*
*
*
*
*
*
j
d
*
### Chart
| Category | control | ADSC NORMA4 | HMEC NORMA4 | MES NORMA4 |
|---|---|---|---|---|
| CD14 | 1.0 | 0.004952615659687678 | 0.00969315068926865 | 0.13908377438831163 |
| CD19 | 1.0 | 0.011560062696443463 | 0.0006873045610097675 | 0.2014383010290164 |
| CD34 | 1.0 | 0.14420989316526311 | 0.0020229757290963404 | 0.025701557781745103 |
| CD45 | 1.0 | 0.0007964557119594451 | 5.782156429496585e-06 | 0.003922142529967264 |
| HLA-DR | 1.0 | 2.250914921217594e-06 | 3.533857299984347e-05 | 0.00012997169813961165 |NORMA4
### Chart
| Category | ADSC NORMA4 | HMEC NORMA4 | Mes NORMA4 |
|---|---|---|---|
| CD105 | 1.0 | 0.053830964591755015 | 0.07556932047523261 |
| CD73 | 1.0 | 0.011547662481461241 | 0.9927348878007348 |
| CD90 | 1.0 | 0.0015637000471008487 | 0.013562825783096852 |
| CD36 | 1.0 | 0.003737243714068755 | 0.32361437526728215 |
| CD29 | 1.0 | 0.1420637059215612 | 1.7453661218664804 |
| CD44 | 1.0 | 0.42222500679489494 | 1.0520469043415115 |
| VIM | 1.0 | 0.028866124830886287 | 1.2013113911626994 |*
fold of control
*
*
*
*
*
*
*
*
fold of control
*
*
*
*
*
*
*
*
*
*
*
*
*
*
*
*
k
e
*
### Chart
| Category | ADSC TRIDUC1 | MES TRIDUC1 |
|---|---|---|
| CD105 | 1.0 | 0.07325615580989454 |
| CD73 | 1.0 | 1.4769599757040153 |
| CD90 | 1.0 | 0.865962874252946 |
| CD36 | 1.0 | 0.13385902866742996 |
| CD29 | 1.0 | 1.4550865894066567 |
| CD44 | 1.0 | 43.45380028130304 |
| VIM | 1.0 | 5.6959387487694855 |
### Chart
| Category | control | ADSC TRIDUC1 | MES TRIDUC1 |
|---|---|---|---|
| CD14 | 1.0 | 0.0032866520196053683 | 0.01557478455158935 |
| CD19 | 1.0 | 0.019493185593356346 | 0.08171682931115928 |
| CD34 | 1.0 | 0.29432554882470613 | 0.008865758427296177 |
| CD45 | 1.0 | 0.0015457852002136582 | 0.0016778923859648428 |
| HLA-DR | 1.0 | 1.117734839727578e-06 | 4.718895637515986e-06 |TRIDUC1
*
*
*
fold of control
fold of control
*
*
*
*
*
*
*
*
*
*
l
f
IFDUC1
### Chart
| Category | ADSC IFDUC1 | HMEC IFDUC1 | MES IFDUC1 |
|---|---|---|---|
| CD105 | 1.0 | 0.08084092253599601 | 0.18173392467560232 |
| CD73 | 1.0 | 0.06966899957527962 | 0.11103958970443217 |
| CD90 | 1.0 | 0.008464703737578143 | 0.031494718646732485 |
| CD36 | 1.0 | 0.0007738481251425368 | 0.3226563740395713 |
| CD29 | 1.0 | 0.04318895621368046 | 0.030372732647076656 |
| CD44 | 1.0 | 3.173940227259131 | 11.271491595737464 |
| VIM | 1.0 | 0.09963201773347279 | 3.761342101030737 |
### Chart
| Category | control | ADSC IFDUC1 | HMEC IFDUC1 | MES IFDUC1 |
|---|---|---|---|---|
| CD14 | 1.0 | 0.005589668711498495 | 0.0074928966243612664 | 0.07735389871980712 |
| CD19 | 1.0 | 0.07148726653330316 | 0.014801204205364146 | 0.21428205148222704 |
| CD34 | 1.0 | 0.7671294397131773 | 0.01106330024802528 | 0.11781276570202691 |
| CD45 | 1.0 | 0.0013954250802517404 | 0.0001925242193189103 | 0.006864982690116927 |
| HLA-DR | 1.0 | 3.816711563731378e-06 | 0.00015397921765741756 | 0.00023776326268229233 |*
*
*
fold of control
fold of control
*
*
*
*
*
*
*
*
*
*
*
*
*
*
*
*
*
*
*
*
*
*
*
*
*
PBC/BEC
MEC ADSC MES
