# Supplementary material for: Selective isolation and characterization of primary cells from normal breast and tumors reveal plasticity of adipose derived stem cells
Source: Breast Cancer Res. 2016 Mar 12;18:32. doi: 10.1186/s13058-016-0688-2 (PMC4788819; doi:10.1186/s13058-016-0688-2)
Supplement: Additional file 3: — Gene expression of invasive and mesenchymal markers in cell isolates. Mammary epithelial cell (MEC), adipose-derived stem cell (ADSC) and mesenchymal cell (MES) primary cell lines all from the same patients (four normal (NORMA1-4) and two breast tumor primary cell lines (invasive inflammatory ductal carcinoma (IFDUC)1, triple-negative ductal carcinoma (TRIDUC)1)) are indicated. Expression results using real-time PCR of genes involved in invasion, MMP2, MMP9 and SNAI1 and markers of the mesenchymal phenotype, N-cadherin (CDH2) and cadherin11 (CDH11) (*p ≤0.05). (PPTX 114 kb) [file 13058_2016_688_MOESM3_ESM.pptx]

## Slide 1
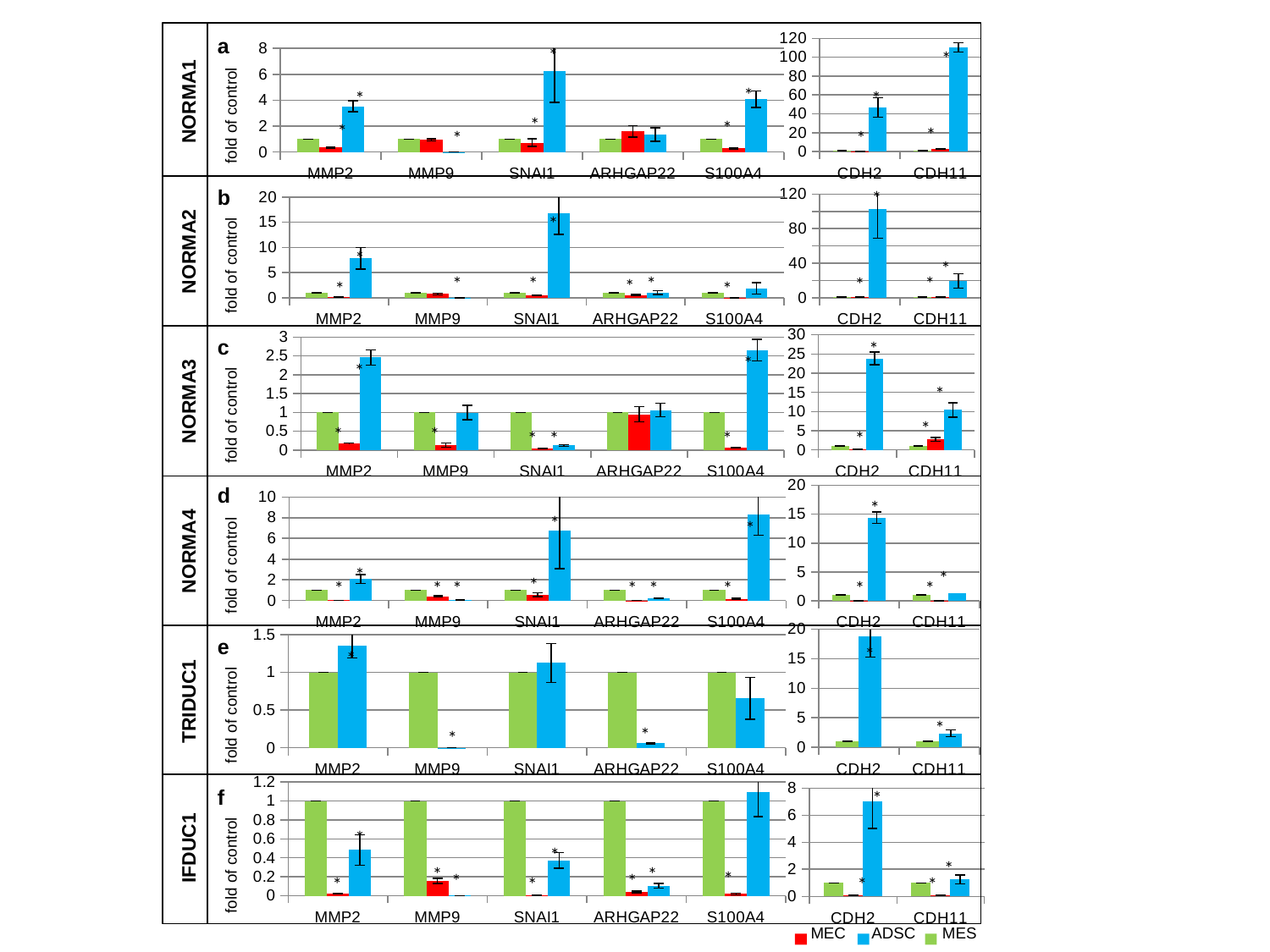

### Chart
| Category | MES NORMA1 | HMEC NORMA1 | ADSC NORMA1 |
|---|---|---|---|
| CDH2 | 1.0 | 0.2012007413634965 | 46.61395971050817 |
| CDH11 | 1.0 | 2.469243363359925 | 110.38414183005365 |a
*
### Chart
| Category | MES NORMA1 | HMEC NORMA1 | ADSC NORMA1 |
|---|---|---|---|
| MMP2 | 1.0 | 0.3586792771625542 | 3.528678223219213 |
| MMP9 | 1.0 | 0.9347959476709354 | 0.005090317095295736 |
| SNAI1 | 1.0 | 0.7238996789625037 | 6.28229468943514 |
| ARHGAP22 | 1.0 | 1.5904906386976947 | 1.344046682837762 |
| S100A4 | 1.0 | 0.281095547472321 | 4.083692046219352 |*
*
NORMA1
*
*
*
*
*
*
fold of control
*
*
b
*
*
### Chart
| Category | MES NORMA2 | HMEC NORMA2 | ADSC NORMA2 |
|---|---|---|---|
| CDH2 | 1.0 | 0.6933112588548725 | 103.13143587335061 |
| CDH11 | 1.0 | 0.6113658729045218 | 19.274647447285556 |
### Chart
| Category | MES NORMA2 | HMEC NORMA2 | ADSC NORMA2 |
|---|---|---|---|
| MMP2 | 1.0 | 0.19172631960531614 | 7.813010136110766 |
| MMP9 | 1.0 | 0.7659822433886027 | 0.003298890946678229 |
| SNAI1 | 1.0 | 0.51103050650521 | 16.710332542431534 |
| ARHGAP22 | 1.0 | 0.5361890400736447 | 1.022251339782368 |
| S100A4 | 1.0 | 0.020936357960761213 | 1.8496204139467303 |NORMA2
*
*
*
*
fold of control
*
*
*
*
*
*
### Chart
| Category | MES NORMA3 | HMEC NORMA3 | ADSC NORMA3 |
|---|---|---|---|
| CDH2 | 1.0 | 0.09513179243608454 | 23.816790807834124 |
| CDH11 | 1.0 | 2.7564383839432653 | 10.407901837352675 |
### Chart
| Category | MES NORMA3 | HMEC NORMA3 | ADSC NORMA3 |
|---|---|---|---|
| MMP2 | 1.0 | 0.18515595147335126 | 2.4611851463684418 |
| MMP9 | 1.0 | 0.12700890869458137 | 0.9952374246931711 |
| SNAI1 | 1.0 | 0.04742355979651595 | 0.12018255993954474 |
| ARHGAP22 | 1.0 | 0.9501449681509101 | 1.0620944381012911 |
| S100A4 | 1.0 | 0.05703014351958019 | 2.652579475410605 |
c
*
*
*
*
NORMA3
*
*
*
*
fold of control
*
*
*
### Chart
| Category | MES NORMA4 | HMEC NORMA4 | ADSC KB |
|---|---|---|---|
| CDH2 | 1.0 | 0.021396334263906625 | 14.362256834954659 |
| CDH11 | 1.0 | 0.011062854033563413 | 1.3854427130852607 |
d
### Chart
| Category | MES NORMA4 | HMEC NORMA4 | ADSC KB |
|---|---|---|---|
| MMP2 | 1.0 | 0.028667051247804207 | 2.0754424116349983 |
| MMP9 | 1.0 | 0.4073357160314899 | 0.03191878859265302 |
| SNAI1 | 1.0 | 0.567115372695228 | 6.726308660705205 |
| ARHGAP22 | 1.0 | 0.01336168239197809 | 0.2376474410472864 |
| S100A4 | 1.0 | 0.17202588849618197 | 8.307714257206948 |*
*
NORMA4
*
*
*
*
fold of control
*
*
*
*
*
*
*
*
### Chart
| Category | MES TRIDUC1 | ADSC TRIDUC1 |
|---|---|---|
| CDH2 | 1.0 | 18.7379651072025 |
| CDH11 | 1.0 | 2.3522311541683427 |
### Chart
| Category | MES TRIDUC1 | ADSC TRIDUC1 |
|---|---|---|
| MMP2 | 1.0 | 1.3496062052597935 |
| MMP9 | 1.0 | 0.0004215547995545826 |
| SNAI1 | 1.0 | 1.1240035777518596 |
| ARHGAP22 | 1.0 | 0.05946388697573935 |
| S100A4 | 1.0 | 0.6540007504226542 |
e
*
*
TRIDUC1
fold of control
*
*
*
### Chart
| Category | MES IFDUC1 | HMEC IFDUC1 | ADSC IFDUC1 |
|---|---|---|---|
| MMP2 | 1.0 | 0.018783185895266317 | 0.48272133193620115 |
| MMP9 | 1.0 | 0.1553903292066309 | 0.0006859007998430399 |
| SNAI1 | 1.0 | 0.007659544121894788 | 0.37250163498477035 |
| ARHGAP22 | 1.0 | 0.04060635780695703 | 0.10373118185229095 |
| S100A4 | 1.0 | 0.018447025990532145 | 1.0900946727474152 |
### Chart
| Category | MES IFDUC1 | HMEC IFDUC1 | ADSC IFDUC1 |
|---|---|---|---|
| CDH2 | 1.0 | 0.06626923360719726 | 6.993380400115936 |
| CDH11 | 1.0 | 0.08065526391393779 | 1.2518995476373826 |f
*
*
IFDUC1
*
*
*
fold of control
*
*
*
*
*
*
*
*
MEC ADSC MES
