# Supplementary material for: Selective isolation and characterization of primary cells from normal breast and tumors reveal plasticity of adipose derived stem cells
Source: Breast Cancer Res. 2016 Mar 12;18:32. doi: 10.1186/s13058-016-0688-2 (PMC4788819; doi:10.1186/s13058-016-0688-2)
Supplement: Additional file 4: — Gene expression of typical epithelial cell markers in cell isolates. Mammary epithelial cell (MEC), adipose-derived stem cell (ADSC) and mesenchymal cell (MES) primary cell lines all from the same patients (four normal (NORMA1-4) and the invasive inflammatory ductal carcinoma (IFDUC)1 breast tumor primary cell line) are indicated. Expression results are shown for a range of typical markers for characterization of epithelial cells, the proliferation marker MKI67 and markers of basal myoepithelial mammary cells (ACTA1, ACTA2, CNN1, CD10) using real-time PCR (*p ≤0.05). (PPTX 132 kb) [file 13058_2016_688_MOESM4_ESM.pptx]

## Slide 1
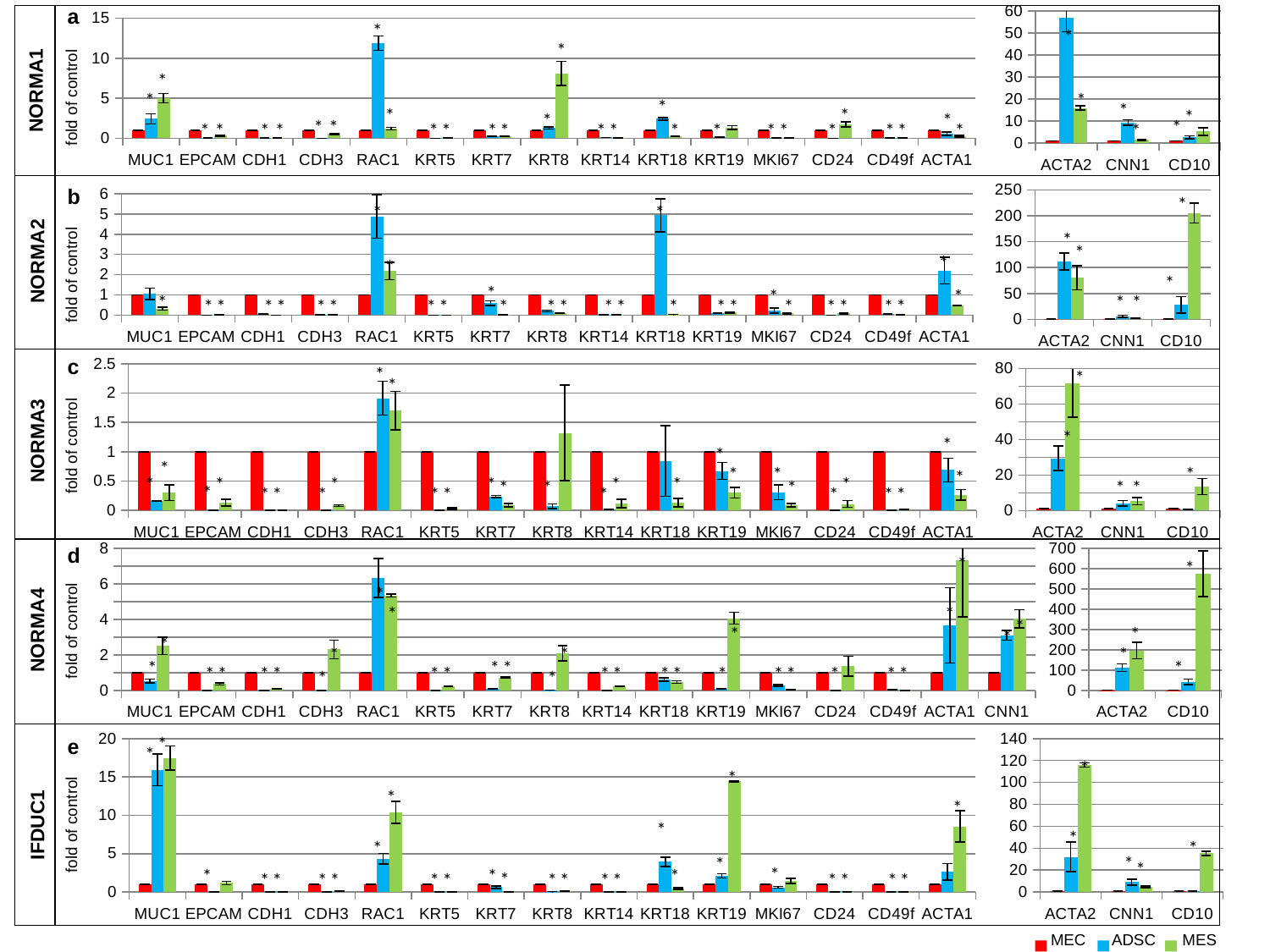

a
### Chart
| Category | HMEC NORMA1 | ADSC NORMA1 | MES NORMA1 |
|---|---|---|---|
| ACTA2 | 1.0 | 56.710446440635394 | 15.878854866563046 |
| CNN1 | 1.0 | 9.321713200948682 | 1.4343414109291084 |
| CD10 | 1.0 | 2.586666280690111 | 5.260406544433412 |
### Chart
| Category | HMEC NORMA1 | ADSC NORMA1 | MES NORMA1 |
|---|---|---|---|
| MUC1 | 1.0 | 2.4309231552916946 | 5.005125029725071 |
| EPCAM | 1.0 | 0.011454057227278708 | 0.33157065420235127 |
| CDH1 | 1.0 | 0.0278064522912063 | 0.033313220463586625 |
| CDH3 | 1.0 | 0.00611241519986247 | 0.5256627912016483 |
| RAC1 | 1.0 | 11.887617455743275 | 1.2147390814828347 |
| KRT5 | 1.0 | 5.347578552643501e-06 | 0.04635350136732477 |
| KRT7 | 1.0 | 0.20217395242015032 | 0.20096054532605465 |
| KRT8 | 1.0 | 1.2846257679899724 | 8.104224432373732 |
| KRT14 | 1.0 | 0.10694302378043437 | 0.04911337546207619 |
| KRT18 | 1.0 | 2.4169969412156447 | 0.22959208894484728 |
| KRT19 | 1.0 | 0.14159477444770965 | 1.3250927557112104 |
| MKI67 | 1.0 | 0.028836923109723186 | 0.023023582067275297 |
| CD24 | 1.0 | 0.0008062984190370104 | 1.726624039389982 |
| CD49f | 1.0 | 0.031188366869764547 | 0.01593010885807799 |
| ACTA1 | 1.0 | 0.5638382139688166 | 0.2562378718752417 |
*
*
*
*
*
*
NORMA1
fold of control
*
*
*
*
*
*
*
*
*
*
*
*
*
*
*
*
*
*
*
*
*
*
*
*
*
*
*
*
*
### Chart
| Category | HMEC NORMA2 | ADSC NORMA2 | MES NORMA2 |
|---|---|---|---|
| MUC1 | 1.0 | 1.045037450201864 | 0.313554847192641 |
| EPCAM | 1.0 | 0.004070677938207299 | 0.02958637333675355 |
| CDH1 | 1.0 | 0.04446581858617943 | 0.002165644518685274 |
| CDH3 | 1.0 | 0.006225630087741479 | 0.03236199942642204 |
| RAC1 | 1.0 | 4.873589418155688 | 2.178671518392433 |
| KRT5 | 1.0 | 2.0358558970450425e-05 | 0.004161542178957177 |
| KRT7 | 1.0 | 0.5999727612583842 | 0.025256788193156656 |
| KRT8 | 1.0 | 0.2034812585418129 | 0.09945566909824978 |
| KRT14 | 1.0 | 0.008044601995434305 | 0.009073034056024826 |
| KRT18 | 1.0 | 4.939893836193881 | 0.03224037355483483 |
| KRT19 | 1.0 | 0.10061875564811625 | 0.12555995317482613 |
| MKI67 | 1.0 | 0.23155972597235688 | 0.0959554908530355 |
| CD24 | 1.0 | 0.00021798459383444522 | 0.08851411110341219 |
| CD49f | 1.0 | 0.059149998300749185 | 0.014670050242762854 |
| ACTA1 | 1.0 | 2.1979385321866705 | 0.4830224703104271 |
b
### Chart
| Category | HMEC NORMA2 | ADSC NORMA2 | MES NORMA2 |
|---|---|---|---|
| ACTA2 | 1.0 | 111.79651998472097 | 80.44022190815319 |
| CNN1 | 1.0 | 6.259269108724393 | 2.592813430741415 |
| CD10 | 1.0 | 27.86085664526499 | 204.90988912264493 |*
*
*
*
*
*
*
NORMA2
*
*
*
fold of control
*
*
*
*
*
*
*
*
*
*
*
*
*
*
*
*
*
*
*
*
*
*
*
*
*
c
### Chart
| Category | HMEC NORMA3 | ADSC NORMA3 | MES NORMA3 |
|---|---|---|---|
| MUC1 | 1.0 | 0.15862976992996566 | 0.3047485371894368 |
| EPCAM | 1.0 | 0.0023811288111100573 | 0.132342431855 |
| CDH1 | 1.0 | 0.0009519869382376491 | 0.003662327667029804 |
| CDH3 | 1.0 | 0.0007595335330942447 | 0.08327176341888894 |
| RAC1 | 1.0 | 1.9104684724287297 | 1.6989838008149514 |
| KRT5 | 1.0 | 1.1808730151777637e-06 | 0.029619634292763083 |
| KRT7 | 1.0 | 0.2385773186916813 | 0.08738479617040225 |
| KRT8 | 1.0 | 0.07161054936333651 | 1.3200332660824285 |
| KRT14 | 1.0 | 0.014627572022771684 | 0.11660747796641313 |
| KRT18 | 1.0 | 0.8421738300715798 | 0.13476307437157084 |
| KRT19 | 1.0 | 0.6718818495708531 | 0.30241470626985095 |
| MKI67 | 1.0 | 0.30821031806807353 | 0.08713535756727248 |
| CD24 | 1.0 | 0.0033379248054486014 | 0.11065095966399842 |
| CD49f | 1.0 | 0.006834547816544855 | 0.016261737036285223 |
| ACTA1 | 1.0 | 0.6876791278150899 | 0.26557976089998125 |*
*
### Chart
| Category | HMEC NORMA3 | ADSC NORMA3 | MES NORMA3 |
|---|---|---|---|
| ACTA2 | 1.0 | 29.42822579961935 | 71.77520127208071 |
| CNN1 | 1.0 | 4.0847493416995215 | 5.1571592653515586 |
| CD10 | 1.0 | 0.6132932009651019 | 13.541436273493177 |*
*
NORMA3
*
*
fold of control
*
*
*
*
*
*
*
*
*
*
*
*
*
*
*
*
*
*
*
*
*
*
*
*
*
*
*
d
### Chart
| Category | HMEC NORMA4 | ADSC NORMA4 | MES NORMA4 |
|---|---|---|---|
| MUC1 | 1.0 | 0.5416874697813642 | 2.50720515270563 |
| EPCAM | 1.0 | 0.001816677518453689 | 0.3699290176362593 |
| CDH1 | 1.0 | 0.0010615865104377199 | 0.10044056714022838 |
| CDH3 | 1.0 | 0.0011050177535965819 | 2.3142246774544684 |
| RAC1 | 1.0 | 6.340057490335166 | 5.353081637823716 |
| KRT5 | 1.0 | 4.6053388577714765e-05 | 0.2257846232287016 |
| KRT7 | 1.0 | 0.11207681112552594 | 0.7252660791573883 |
| KRT8 | 1.0 | 0.016614750339846676 | 2.0974344222609957 |
| KRT14 | 1.0 | 0.0059408518041301995 | 0.24671315577479982 |
| KRT18 | 1.0 | 0.6169434685175917 | 0.4747473385536818 |
| KRT19 | 1.0 | 0.10931227572114026 | 4.071757746797786 |
| MKI67 | 1.0 | 0.28564197992360624 | 0.05849491914828484 |
| CD24 | 1.0 | 9.973645267350496e-05 | 1.3612968673756078 |
| CD49f | 1.0 | 0.03418908156997835 | 0.008588099904006842 |
| ACTA1 | 1.0 | 3.656885513517159 | 7.324461859840291 |
| CNN1 | 1.0 | 3.099354544578412 | 4.042444806976937 |
### Chart
| Category | HMEC NORMA4 | ADSC NORMA4 | MES NORMA4 |
|---|---|---|---|
| ACTA2 | 1.0 | 112.63981127381658 | 197.53517106546744 |
| CD10 | 1.0 | 42.87973232330538 | 574.83370368931 |
*
*
*
*
*
*
*
*
NORMA4
fold of control
*
*
*
*
*
*
*
*
*
*
*
*
*
*
*
*
*
*
*
*
*
*
*
*
*
*
*
### Chart
| Category | HMEC IFDUC1 | ADSC IFDUC1 | MES IFDUC1 |
|---|---|---|---|
| MUC1 | 1.0 | 15.94446946717745 | 17.456845823062242 |
| EPCAM | 1.0 | 0.007877476986090594 | 1.172866149289013 |
| CDH1 | 1.0 | 0.007184322906662392 | 0.0045747285588763195 |
| CDH3 | 1.0 | 0.002269209639818706 | 0.07225854874314014 |
| RAC1 | 1.0 | 4.320505563096733 | 10.402847902770823 |
| KRT5 | 1.0 | 3.024837387745846e-05 | 0.00430948209168629 |
| KRT7 | 1.0 | 0.6358909496752282 | 0.00363060957230118 |
| KRT8 | 1.0 | 0.06223063825613406 | 0.14016942571867289 |
| KRT14 | 1.0 | 0.002658399419883493 | 0.0003480986962871517 |
| KRT18 | 1.0 | 3.9294019723143627 | 0.44709846950780313 |
| KRT19 | 1.0 | 2.112493270672701 | 14.45390391343041 |
| MKI67 | 1.0 | 0.6185314916509379 | 1.4520524462627395 |
| CD24 | 1.0 | 0.00045451956432007875 | 0.004134994607481857 |
| CD49f | 1.0 | 0.0036023701352817096 | 0.008426390397476632 |
| ACTA1 | 1.0 | 2.6292899873057474 | 8.552228402156032 |*
### Chart
| Category | HMEC IFDUC1 | ADSC IFDUC1 | MES IFDUC1 |
|---|---|---|---|
| ACTA2 | 1.0 | 31.98013318301012 | 115.90423814934915 |
| CNN1 | 1.0 | 9.08511681945709 | 4.601568466858368 |
| CD10 | 1.0 | 1.010546063820818 | 35.29506693339304 |e
*
*
*
*
*
*
*
*
IFDUC1
fold of control
*
*
*
*
*
*
*
*
*
*
*
*
*
*
*
*
*
*
*
*
*
*
*
MEC ADSC MES
