# Supplementary material for: Selective isolation and characterization of primary cells from normal breast and tumors reveal plasticity of adipose derived stem cells
Source: Breast Cancer Res. 2016 Mar 12;18:32. doi: 10.1186/s13058-016-0688-2 (PMC4788819; doi:10.1186/s13058-016-0688-2)
Supplement: Additional file 5: — Endothelial-like differentiation of adipose-derived stem cells (ADSC) into capillary structures. Endothelial-like differentiation was quantified 5 h after seeding ADSC on Matrigel®. a X axis represents the tube length in mm for each capillary side cell structure. b X axis represents the number of completely closed capillary loop structures. c X axis represents the area in mm2 for completely closed and open capillary structures. a-c Y axis represents the origin of the ADSC (triple-negative ductal carcinoma (TRIDUC)1, invasive inflammatory ductal carcinoma (IFDUC)1 or normal breast (NORMA)4). Quantification was performed in three wells (total area 0.125 cm2/well). (PPTX 52 kb) [file 13058_2016_688_MOESM5_ESM.pptx]

## Slide 1
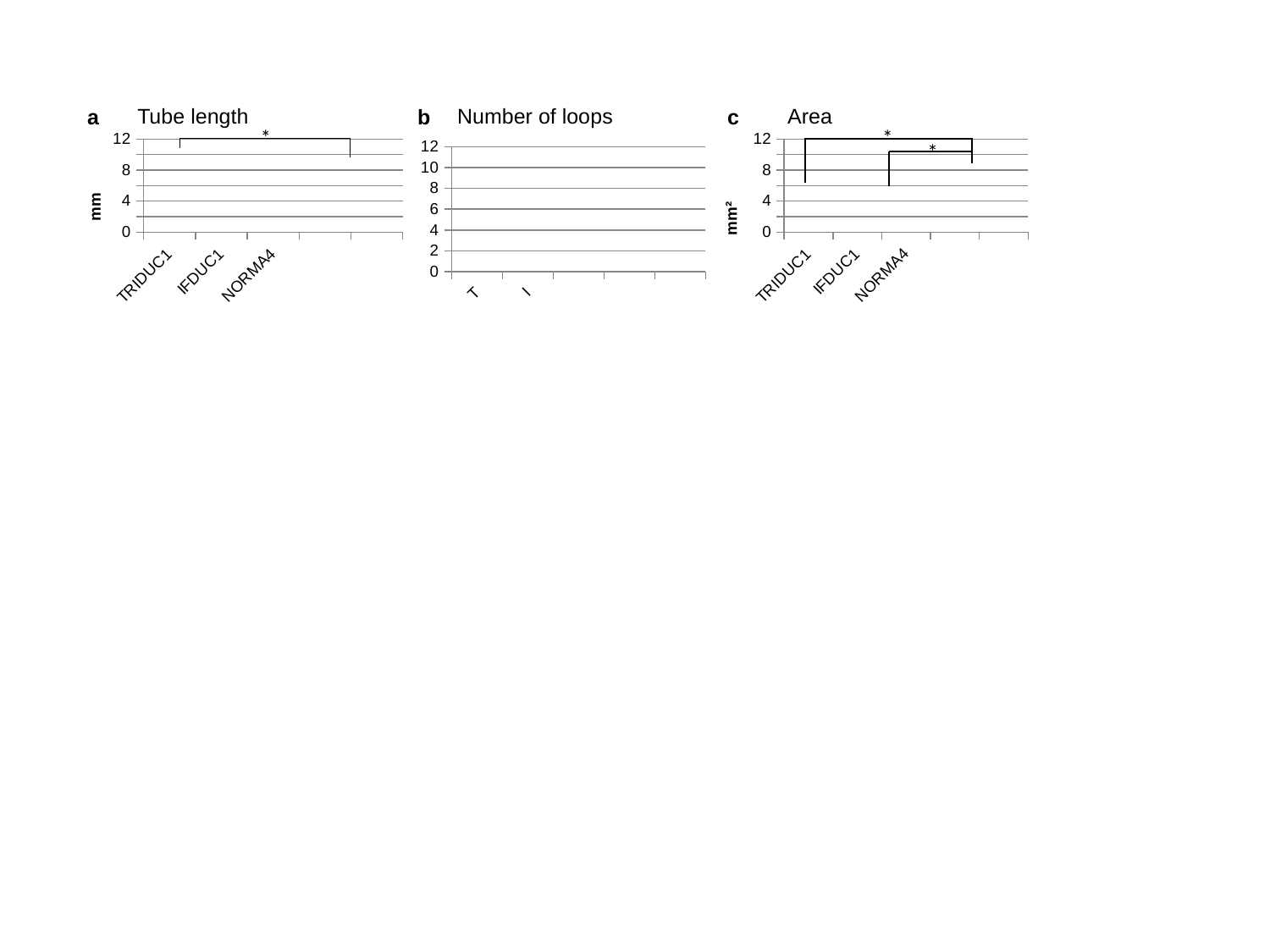

Tube length
### Chart
| Category | 1w |
|---|---|
| TRIDUC1 | 191.33333333333334 |
| IFDUC1 | 160.33333333333334 |
| NORMA4 | 167.0 |Number of loops
Area
a
b
c
*
*
### Chart
| Category | 1w |
|---|---|
| TRIDUC1 | 89.43530633333333 |
| IFDUC1 | 79.48677799999999 |
| NORMA4 | 79.44658333333332 |
### Chart
| Category | 1w |
|---|---|
| TRIDUC1 | 4.442064834911863 |
| IFDUC1 | 4.627472594095097 |
| NORMA4 | 6.170789196287687 |*
